# Supplementary figures and images for: Single-cell sequencing reveals lineage-specific dynamic genetic regulation of gene expression during human cardiomyocyte differentiation
Source: PLoS Genet. 2022 Jan 21;18(1):e1009666. doi: 10.1371/journal.pgen.1009666 (PMC8809621; doi:10.1371/journal.pgen.1009666)

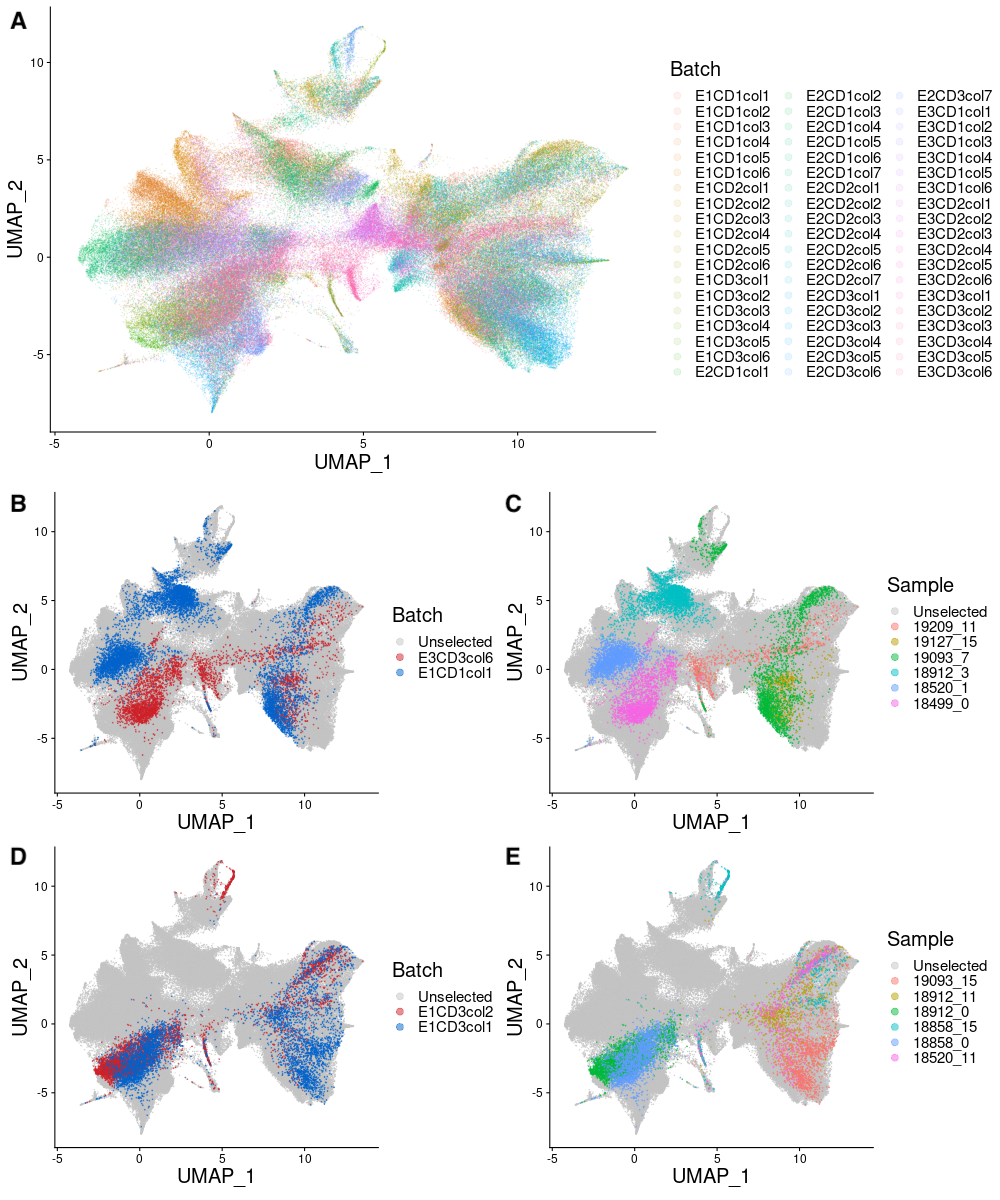

Supplement: S1 Fig — (A) Cells are colored by batch (the experiment, collection day, and collection in which they were collected for sequencing). (B-E) Coloring by batch (B, D) and sample (C, E) shows that apparent batch effects are driven by similarity between cells of the same sample (cell line and differentiation day) within the batch, rather than the overall batch itself. (TIF) [file pgen.1009666.s003.tif]

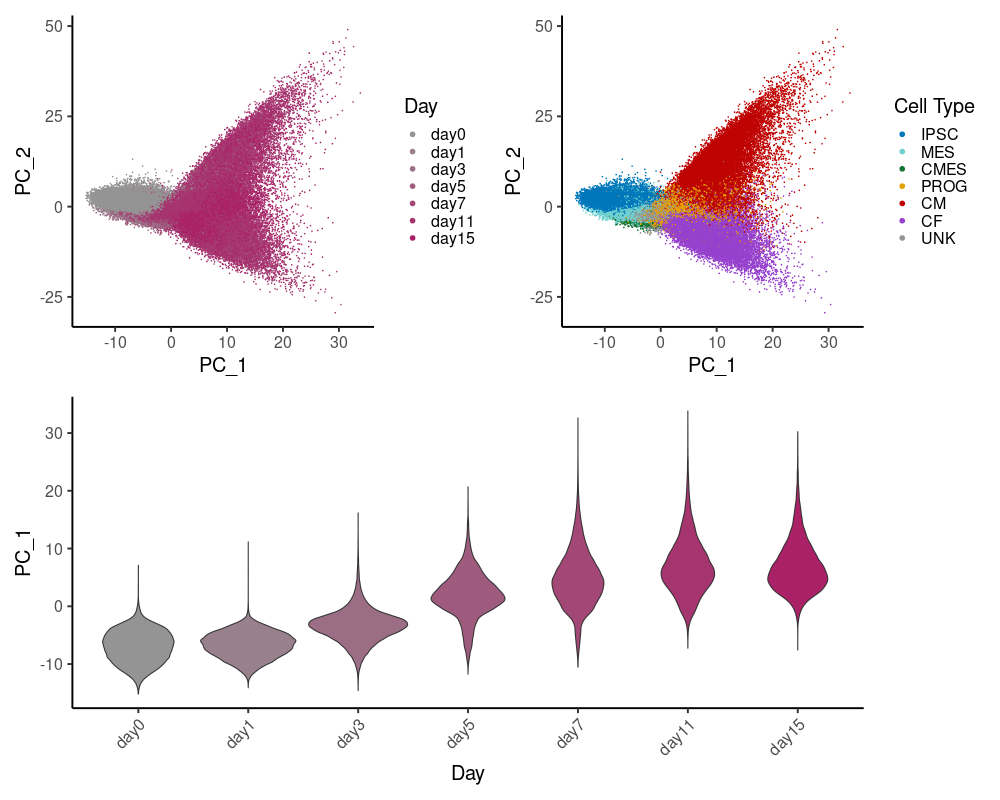

Supplement: S2 Fig — (Top Left) Principal components biplot for single cell data, colored by differentiation day. (Top Right) Principal components biplot for single cell data, colored by cell type. (Bottom) Violin plot of PC1 loadings on each cell, grouped by differentiation day. (TIF) [file pgen.1009666.s004.tif]

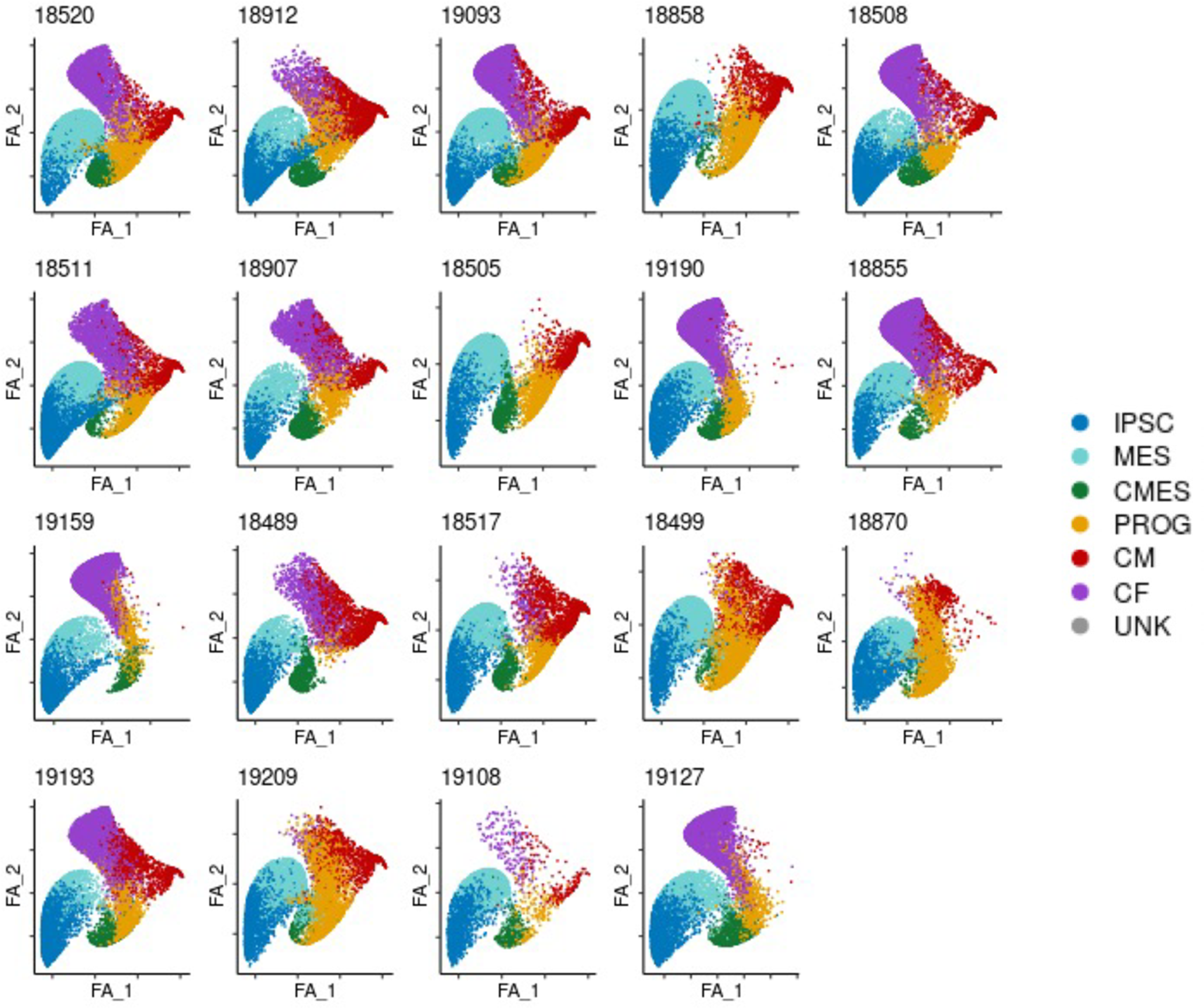

Supplement: S3 Fig — The force atlas embedding which was learned from all cells jointly is shown for each individual cell line, colored by cell type. (TIF) [file pgen.1009666.s005.tif]

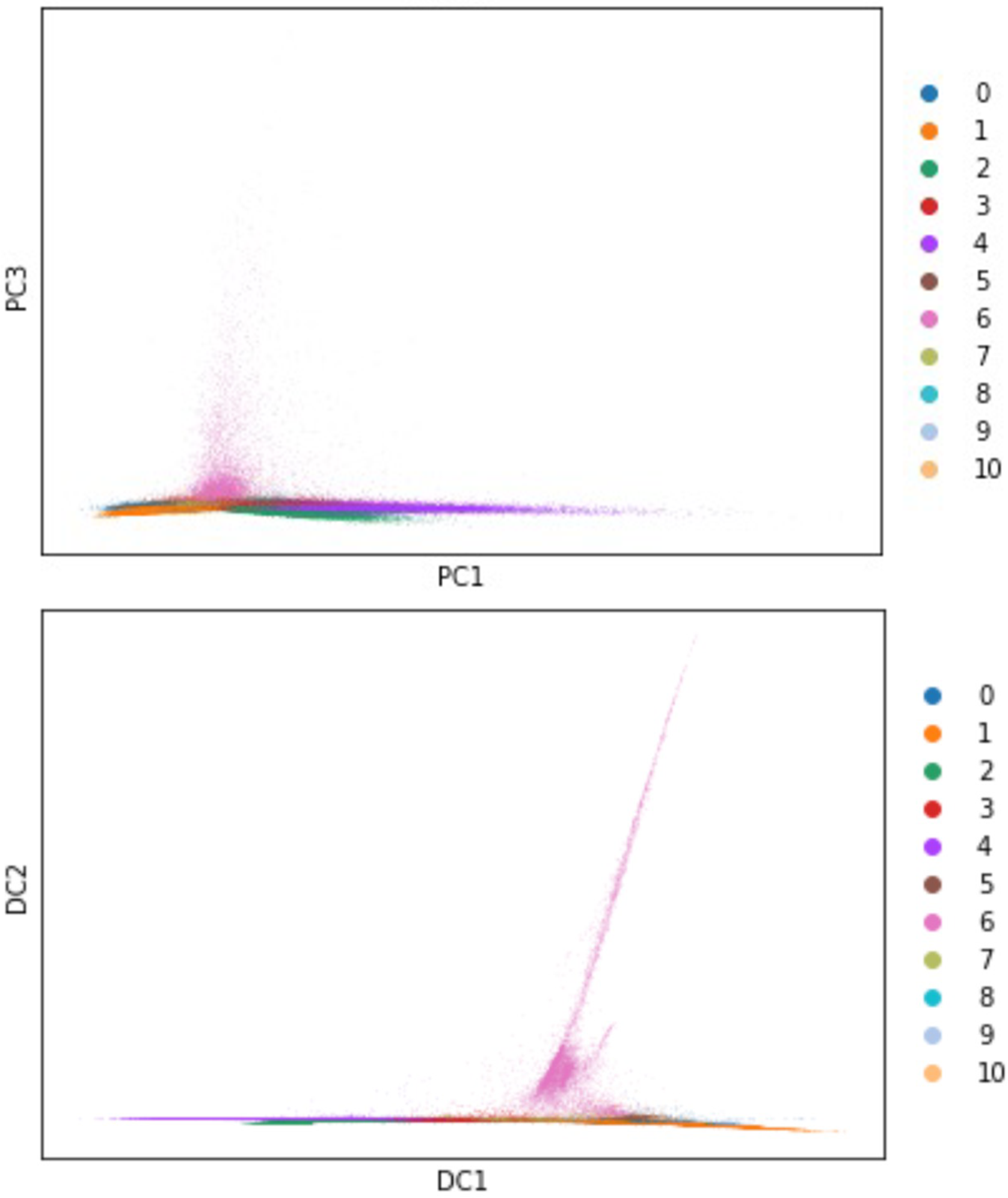

Supplement: S4 Fig — This group of cells which underexpresses cardiac markers from all stages of differentiation and overexpresses endoderm markers such as APOA1 and AFP is picked up by the third principal component (top), and largely drives the variation behind the second diffusion component (bottom). The variation driven by relatively small population of cells interferes with reconstruction of biologically feasible trajectories, and was removed from downstream analysis. (TIF) [file pgen.1009666.s006.tif]

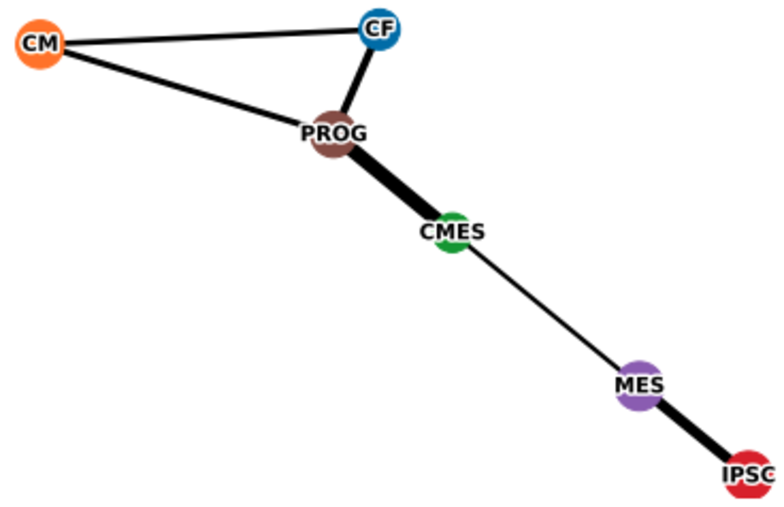

Supplement: S5 Fig — PAGA identifies a bifurcation into cardiomyocyte and cardiac fibroblast cell types after the cardiac progenitor stage. (TIF) [file pgen.1009666.s007.tif]

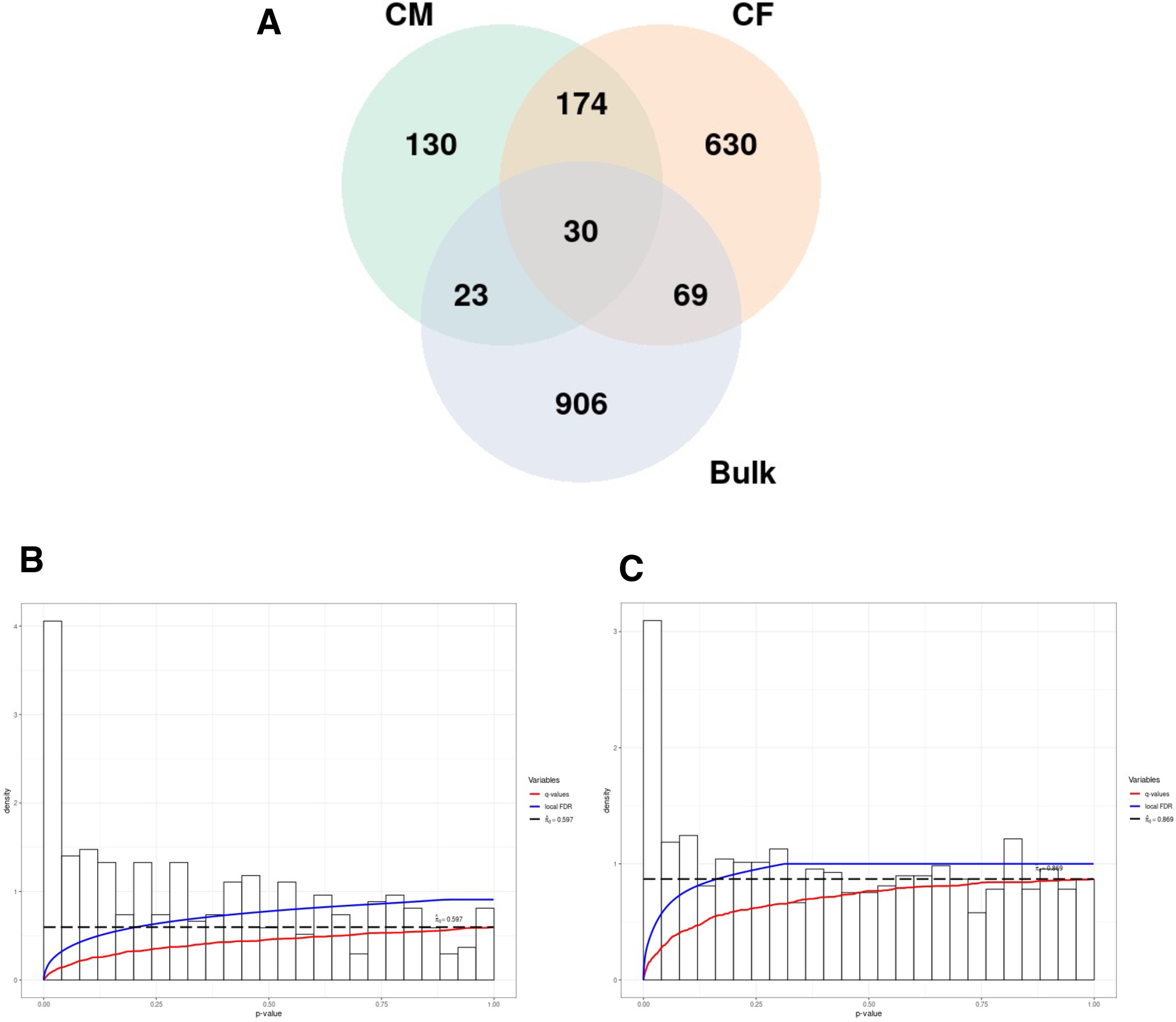

Supplement: S6 Fig — (A) Number of dynamic eGenes that were detected in common between pseudotime-binned cardiomyocyte lineage, pseudotime-binned cardiac fibroblast, and previously collected bulk data. The majority of cardiomyocyte lineage dynamic eGenes overlap with at least one of the other two analyses. (B, C) Replication analysis of pseudobulk dynamic eQTLs in bulk [73]. (B) Distribution of nominal p-values from bulk data for the subset of gene-variant pairs that were identified as a dynamic eQTL in the pseudotime-binned cardiomyocyte lineage (π1 = 0.40). (C) Distribution of nominal p-values from bulk data for the subset of gene-variant pairs that were identified as a dynamic eQTL in the pseudotime-binned cardiac fibroblast lineage (π1 = 0.13). (TIF) [file pgen.1009666.s008.tif]

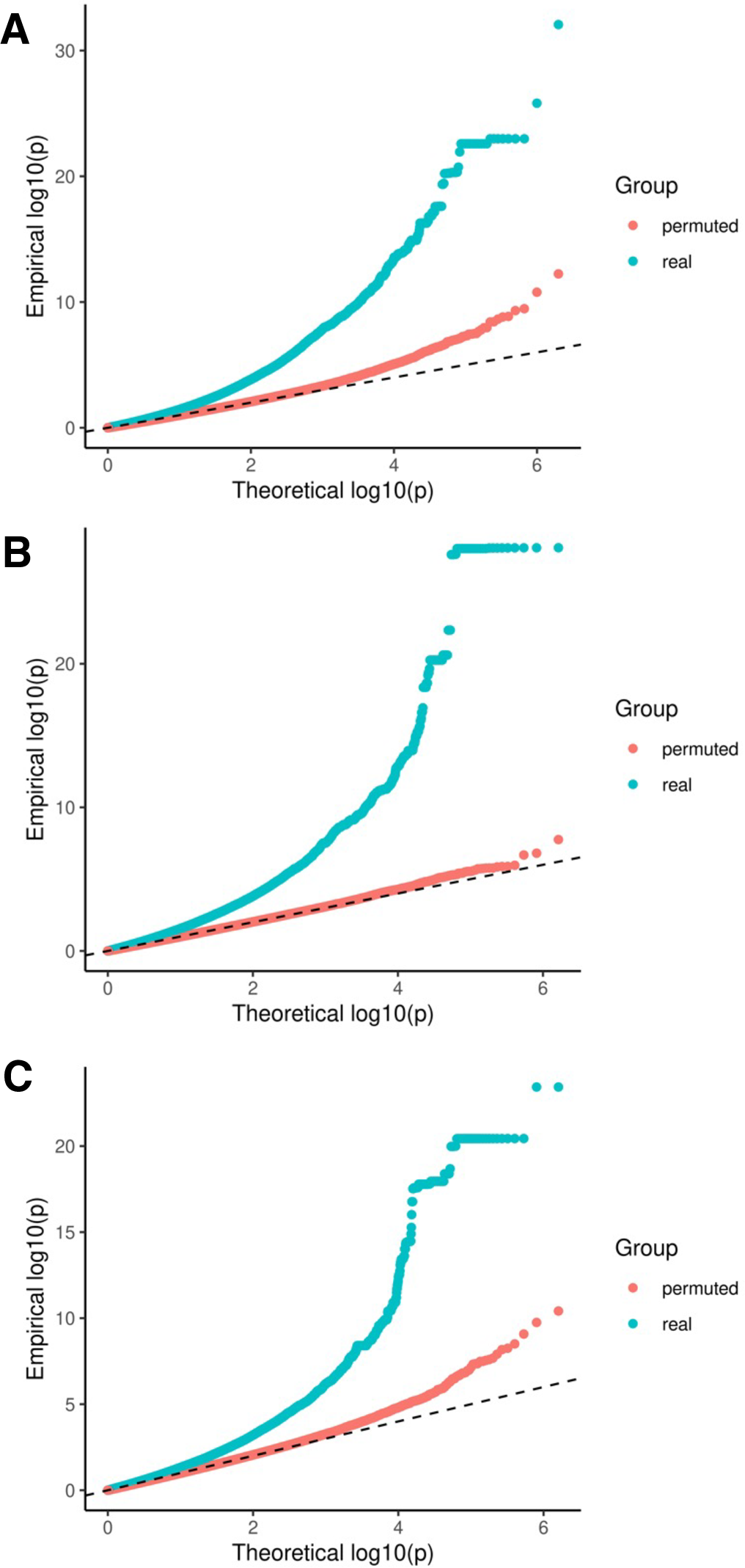

Supplement: S7 Fig — Permutation analyses (see Control Experiments in Materials and Methods) do not suggest substantial inflation in bulk (a), pseudotime-binned cardiomyocyte-subset pseudobulk (b), or pseudotime-binned cardiac fibroblast-subset pseudobulk (c). The p-values from this study are shown in blue, while those obtained from a permutation test are shown in red. (TIF) [file pgen.1009666.s009.tif]

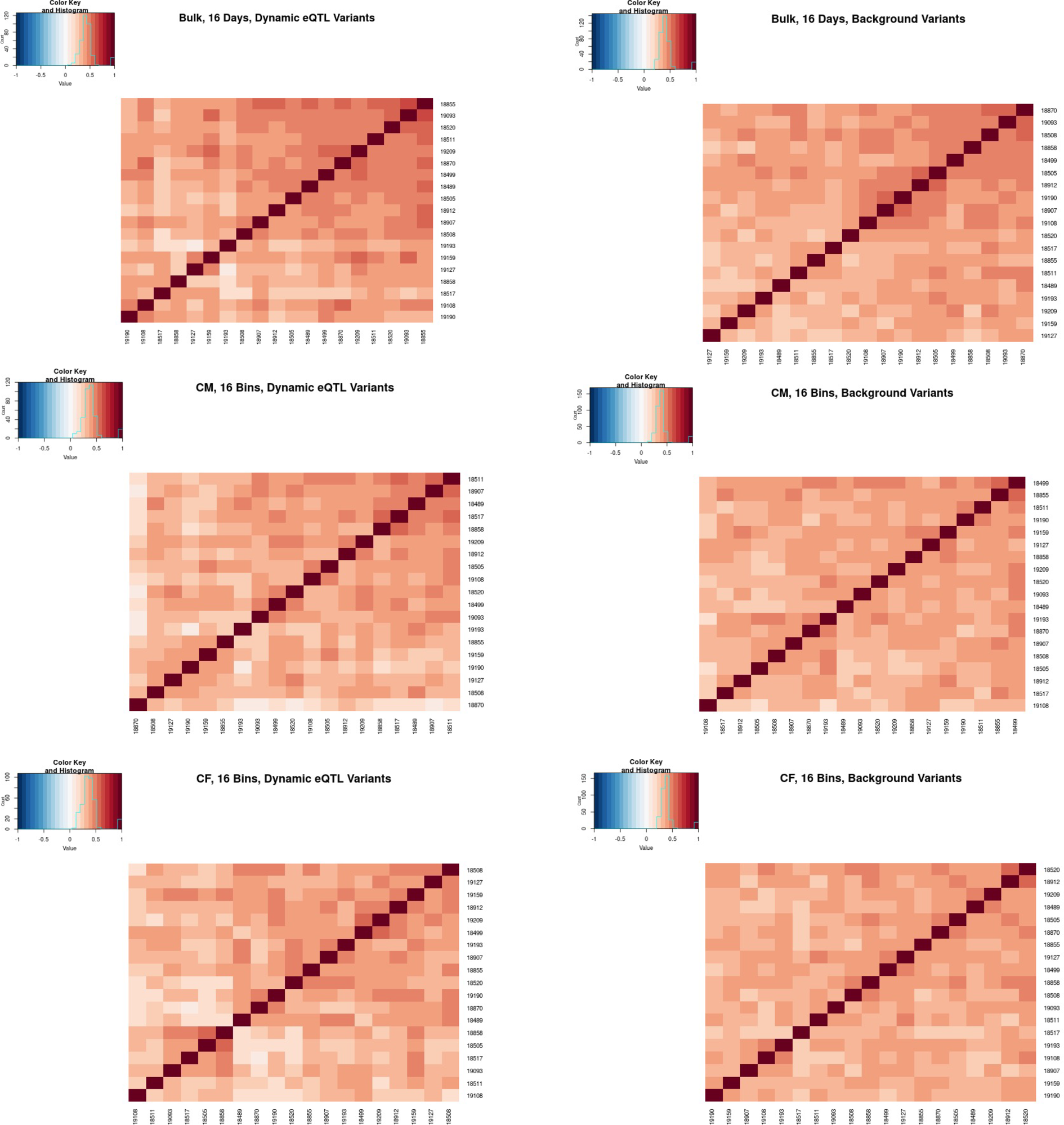

Supplement: S8 Fig — In order to check whether broad cell line differences are driving false positive dynamic eQTLs, we compared genetic correlation among the top 200 linear dynamic eQTLs for bulk (top, left), and both pseudobulk lineages, cardiomyocyte (middle, left) and cardiac fibroblast (bottom, left), to genetic correlation among a set of background variants within 50kb of a gene, and matched for minor allele frequency (right). (TIF) [file pgen.1009666.s010.tif]

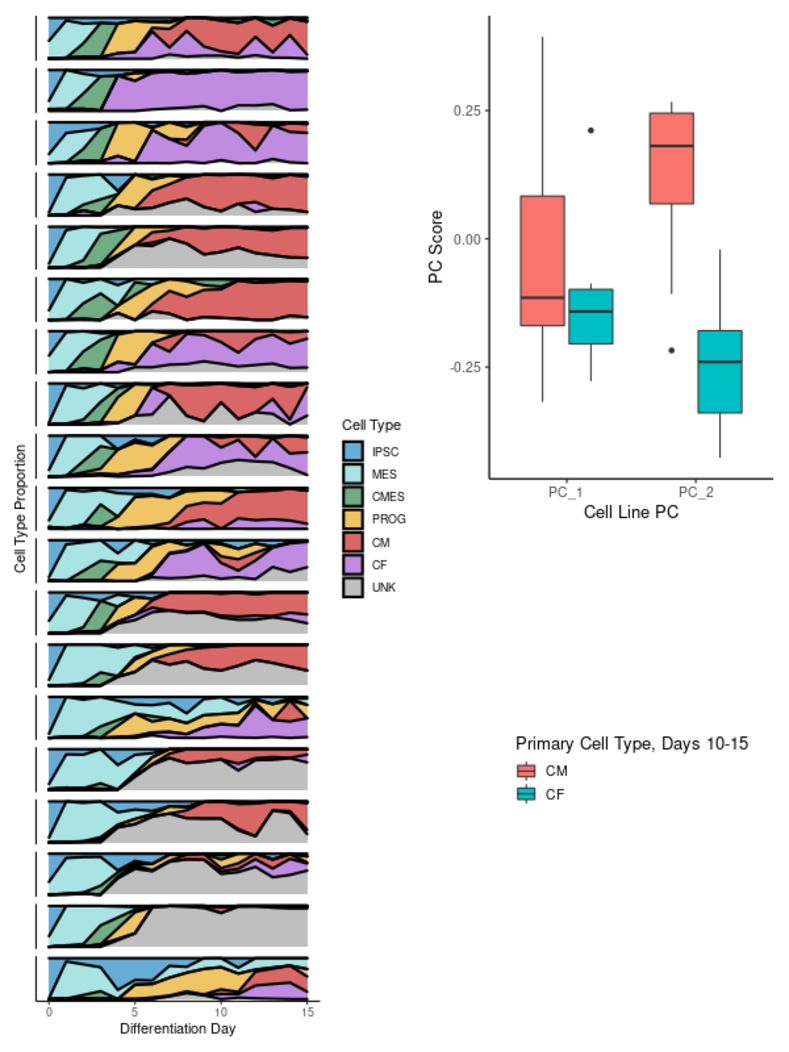

Supplement: S9 Fig — (Left) Inferred cell type proportions in bulk for each of the 19 cell lines, sorted by cell line PC1 loading. Focusing particularly on the proportions of iPSC and mesoderm cells, (blue and teal, respectively), it appears that cell line PC1 is picking up on differentiation speed, with cell lines with a higher PC1 score (lower subplots) differentiating slower than cells with a lower PC1 score. (Right) The second cell line PC score appears to separate cell lines based on their terminal cell type preference, cardiomyocyte or cardiac fibroblast, as defined by the most common cell type among differentiation days 10 to 15. (TIF) [file pgen.1009666.s011.tif]

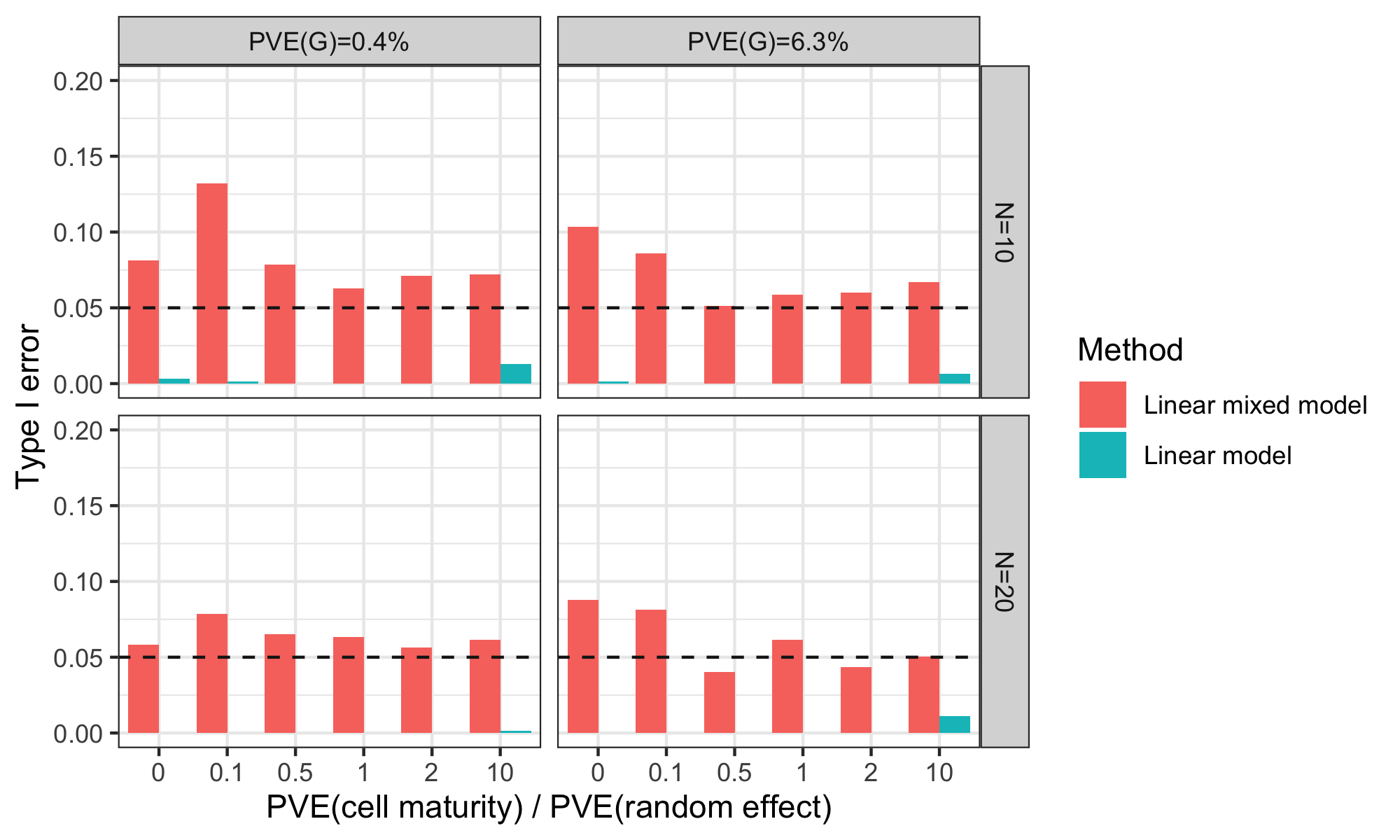

Supplement: S10 Fig — Simulations were performed to examine the impact of selective inference on type I error rates (Simulations to examine type I errors due to ’double dipping’). Under the generative model used, inflated type I error rates (bars exceeding the dashed line) were not observed when testing is performed using a linear model (blue). (TIF) [file pgen.1009666.s012.tif]

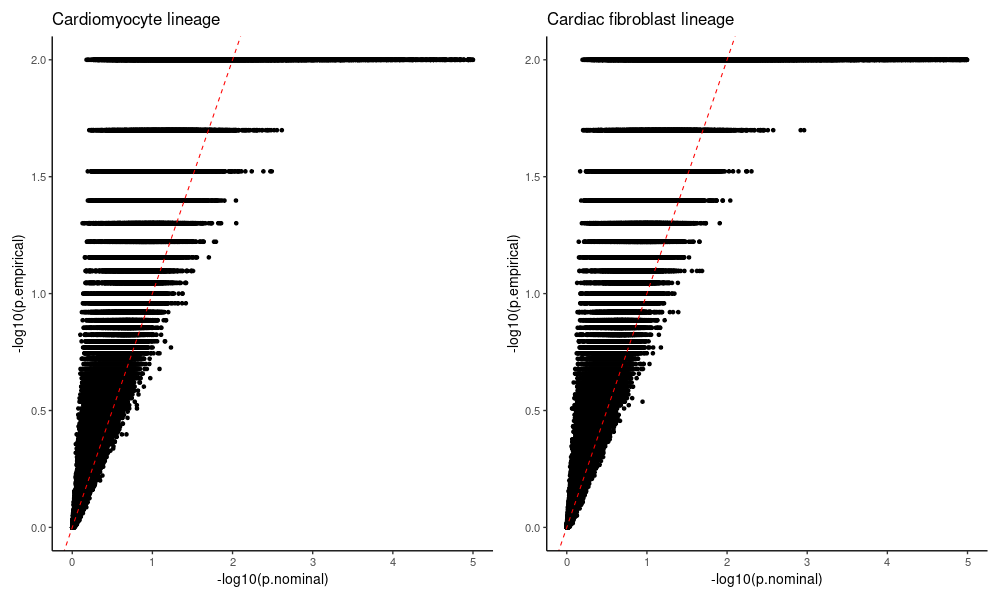

Supplement: S11 Fig — We generated an empirical null distribution by performing dynamic eQTL calling after permuting cell pseudotime upstream of pseudobulk aggregation. We compared the resulting empirical p-values (y-axis) to the nominal p-values from the original analysis (x-axis) in both lineages. We did not find evidence of inflation in the nominal p-values from the original analysis (instead, the contrast between the distributions suggests the nominal p-values may be overly conservative). (TIF) [file pgen.1009666.s013.tif]

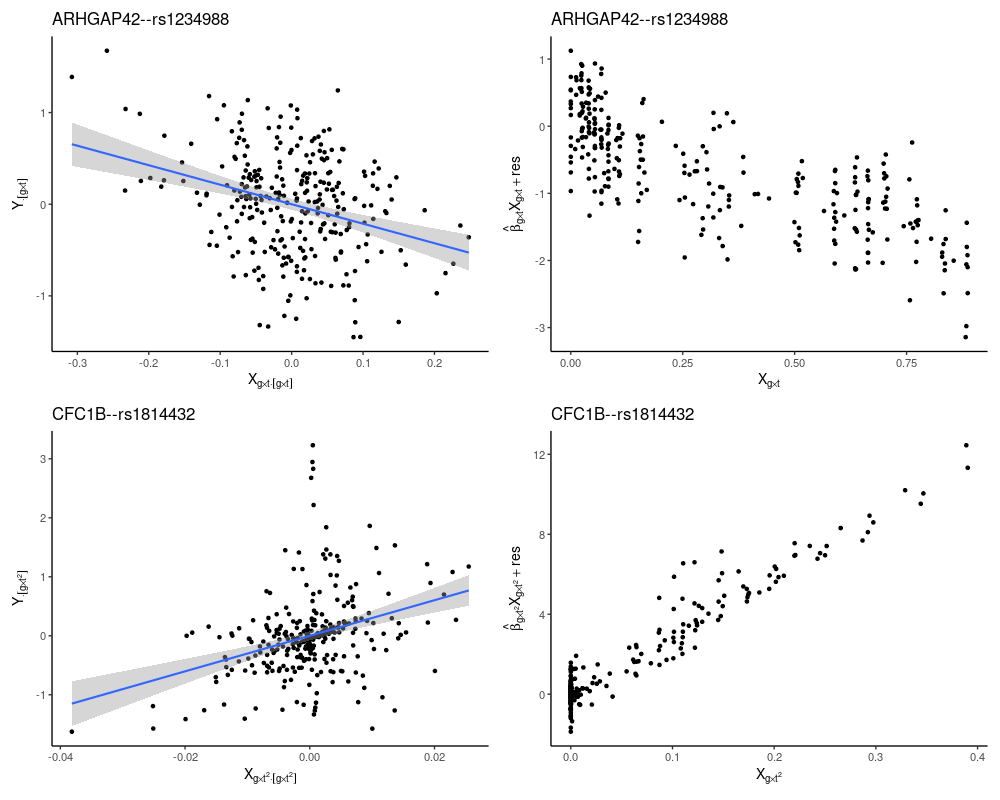

Supplement: S12 Fig — (Top left) Linear dynamic eQTL, partial regression plot. For the linear dynamic eQTL example shown in Fig 3A (βg*t^=−2.13,p=1.64*10−7,q=9.7*10−4), we obtained the residuals from regressing expression on all independent variables except the genotype * pseudotime interaction term (y-axis), and plotted these against the residuals from regressing the interaction term itself on all other independent variables (x-axis). The slope of the line shown measures the effect of the interaction between genotype and pseudotime after controlling for all other independent variables. (Top right) Linear dynamic eQTL, partial residuals plot. On the y-axis, Xg*tβg*t+res, where Xg*t is genotype*time for a cell line/pseudotime bin pseudobulk sample, βg*t is the estimated coefficient for the genotype*time interaction term, and res are the residuals from the fitted linear dynamic eQTL model. On the x-axis is Xg*t. (Bottom) Similar partial regression and partial residuals plots (respectively) for the nonlinear dynamic eQTL shown in Fig 3C (βg*t2^=30.1,p=1.26*10−8,q=1.3*10−3), where the interaction term of interest is between genotype and pseudotime squared. (TIF) [file pgen.1009666.s014.tif]

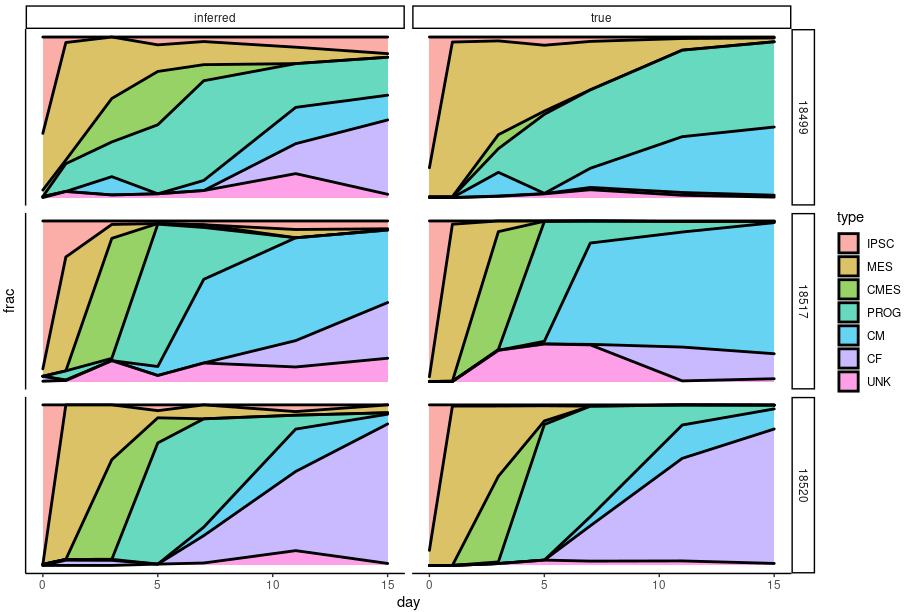

Supplement: S13 Fig — Assessment of CIBERSORTx performance in pseudobulk, where ’ground truth’ is available. CIBERSORTx-estimated cell type proportions from differentiation day-binned pseudobulk data for three cell lines is shown at left (’inferred’), compared to true cell type proportions (’true’, right), as determined by the cell type annotation approach described in the supplement. (TIF) [file pgen.1009666.s015.tif]

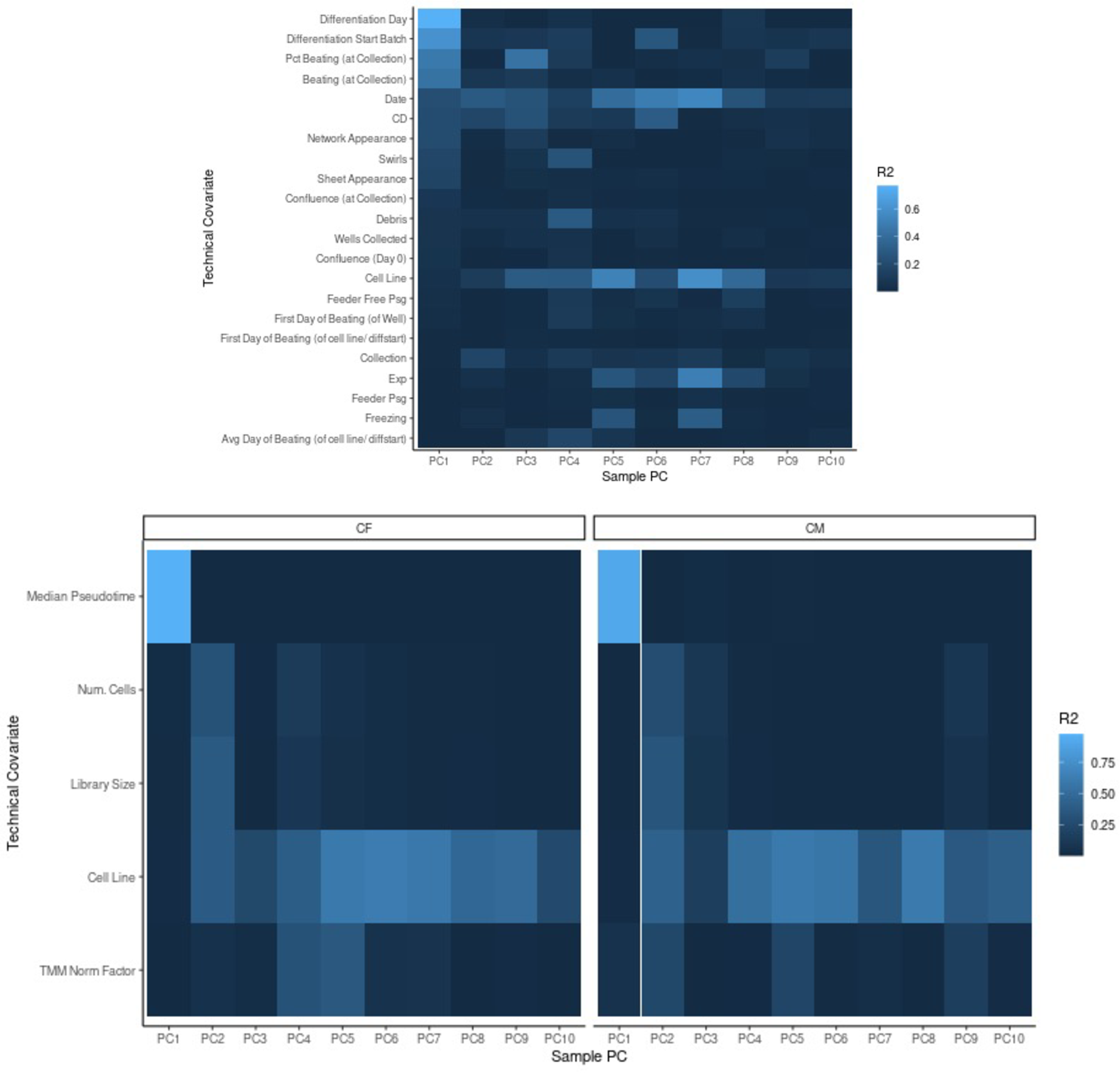

Supplement: S14 Fig — (a) Variance explained of each gene expression principal component (1–10) for pseudobulk samples aggregated by cell line and differentiation day using recorded covariates, including: percent cells beating (visually assessed), differentiation day, collection day, culture confluence, cell morphology (visually assessed), and cellular debris. (b) Variance explained of principal components for pseudobulk samples aggregated by cell line pseudotime bin for cardiac fibroblast (CF, left) and cardiomyocyte (CM, right) lineages. Technical covariates shown are cell line, library size, median pseudotime, number of cells, and the normalization factor used for TMM normalization, from the edgeR package (see Materials and Methods). (TIF) [file pgen.1009666.s016.tif]

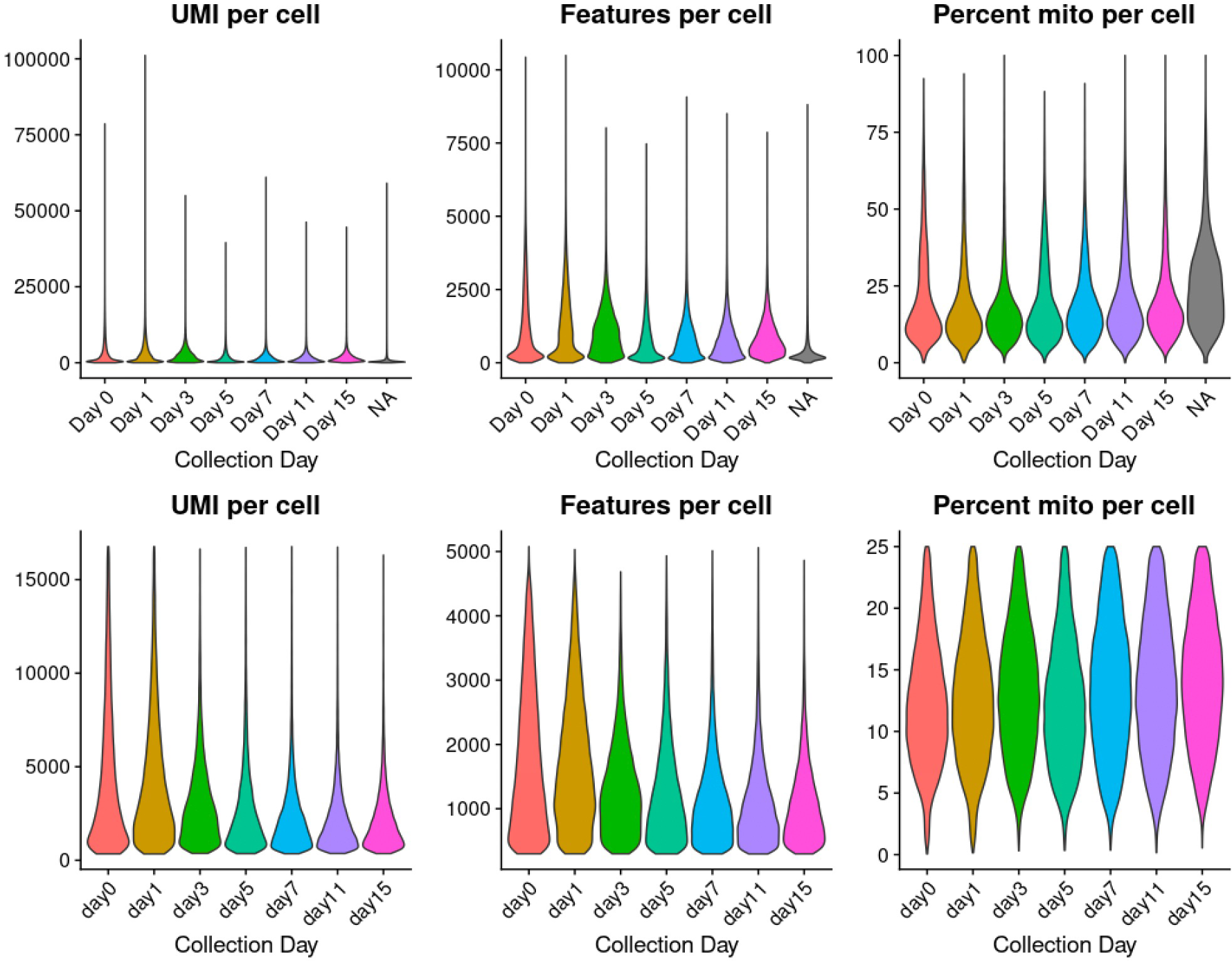

Supplement: S15 Fig — Distribution of the number of Unique Molecular Identifiers (UMIs) per cell, number of genes per cell, and the percent mitochondrial reads per cell in full single cell dataset, prior to (top row) and after (bottom row) filtering as described in Materials and Methods (RNA-seq quantification). X-axis separated by differentiation day. (TIF) [file pgen.1009666.s017.tif]

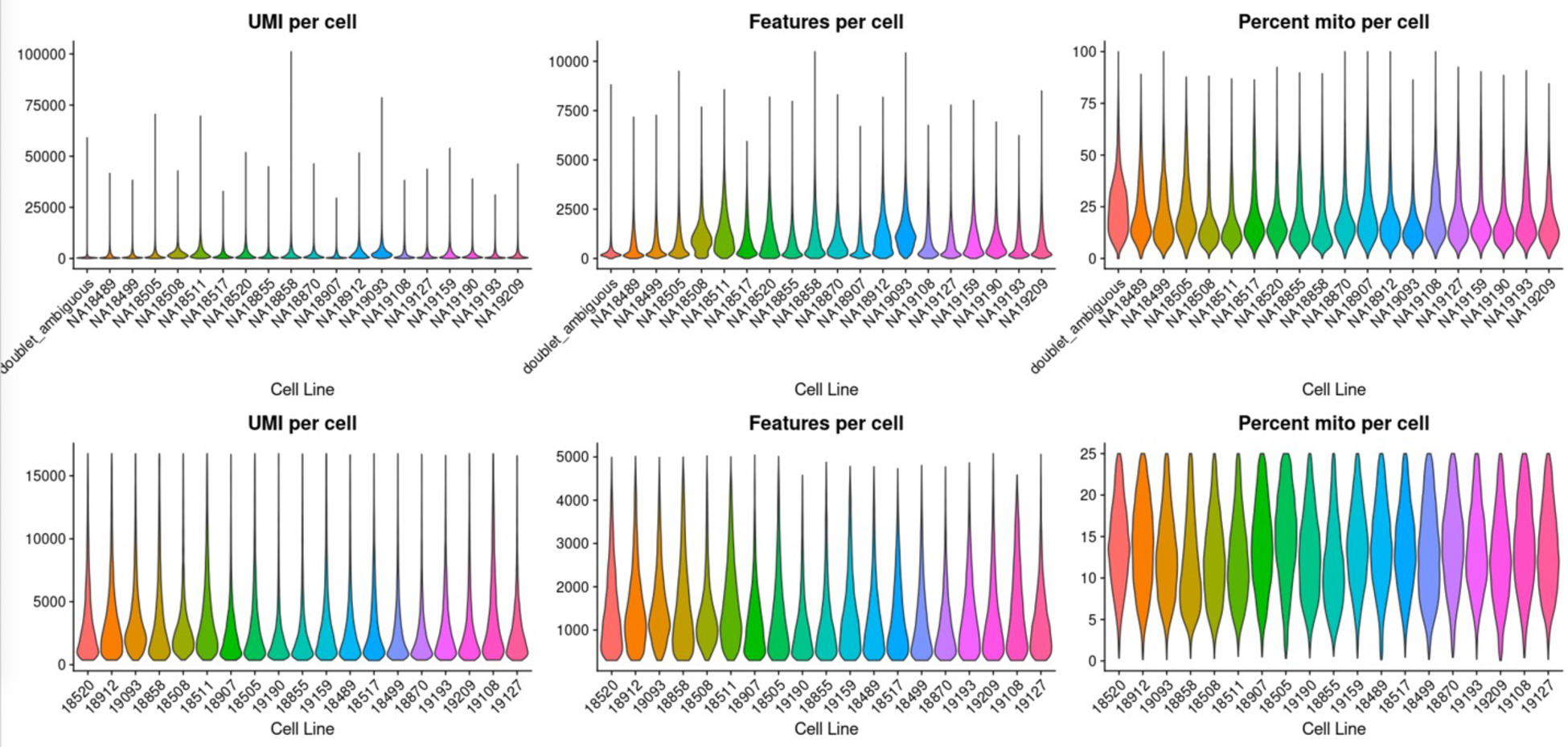

Supplement: S16 Fig — Distribution of the number of Unique Molecular Identifiers (UMIs) per cell, number of genes per cell, and the percent mitochondrial reads per cell in full single cell dataset, prior to (top row) and after (bottom row) filtering as described in Materials and Methods (RNA-seq quantification). X-axis separated by cell line. (TIF) [file pgen.1009666.s018.tif]

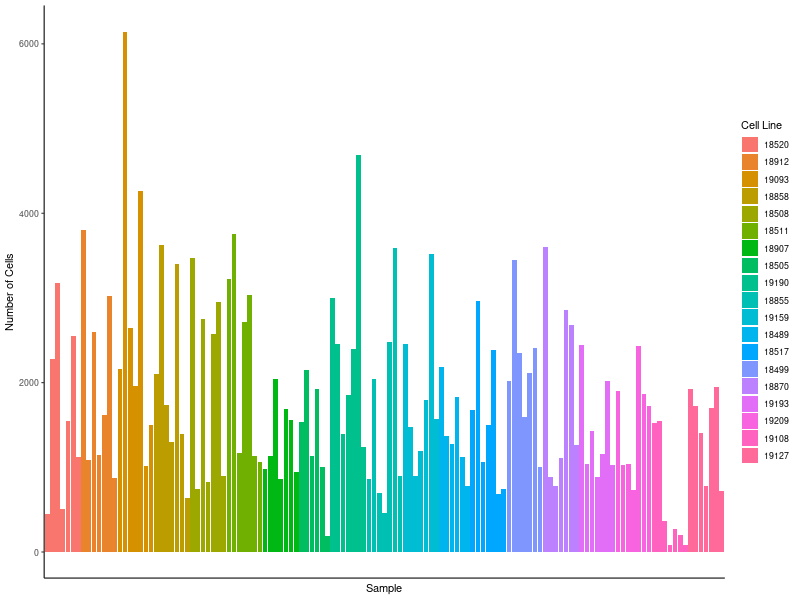

Supplement: S17 Fig — Number of cells per collected sample following filtering described in Materials and Methods (RNA-seq quantification). (TIF) [file pgen.1009666.s019.tif]

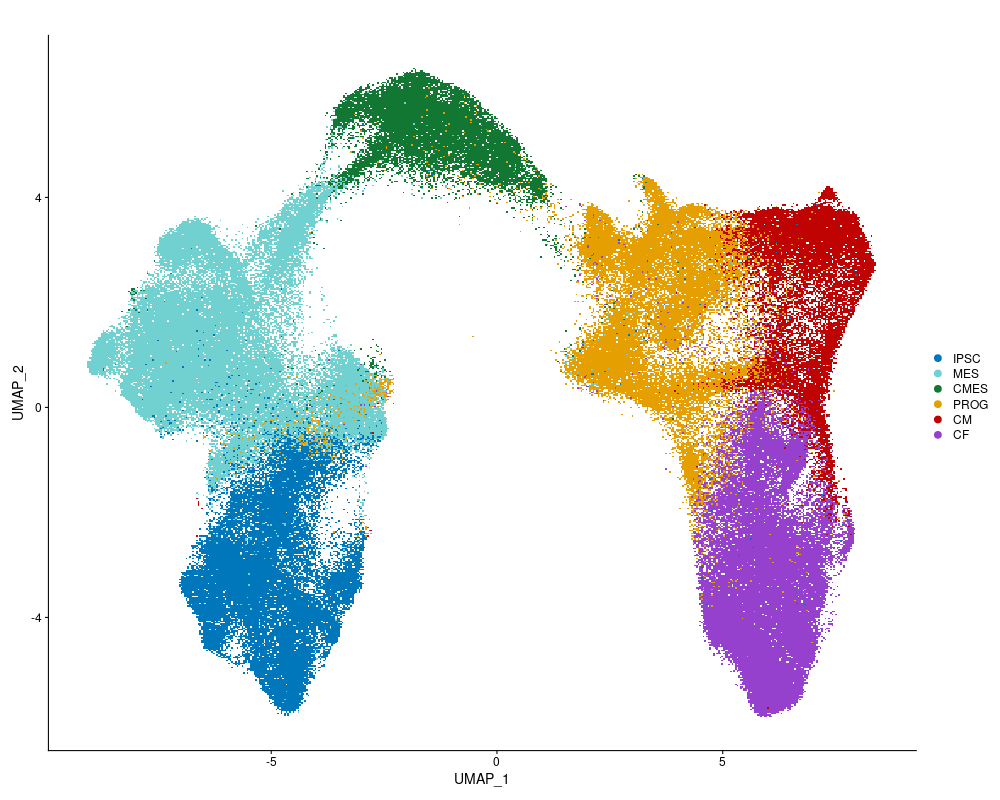

Supplement: S18 Fig — As in Fig 1C, a UMAP embedding of the single cell dataset colored by cell type, except with outlier clusters removed. (TIF) [file pgen.1009666.s020.tif]

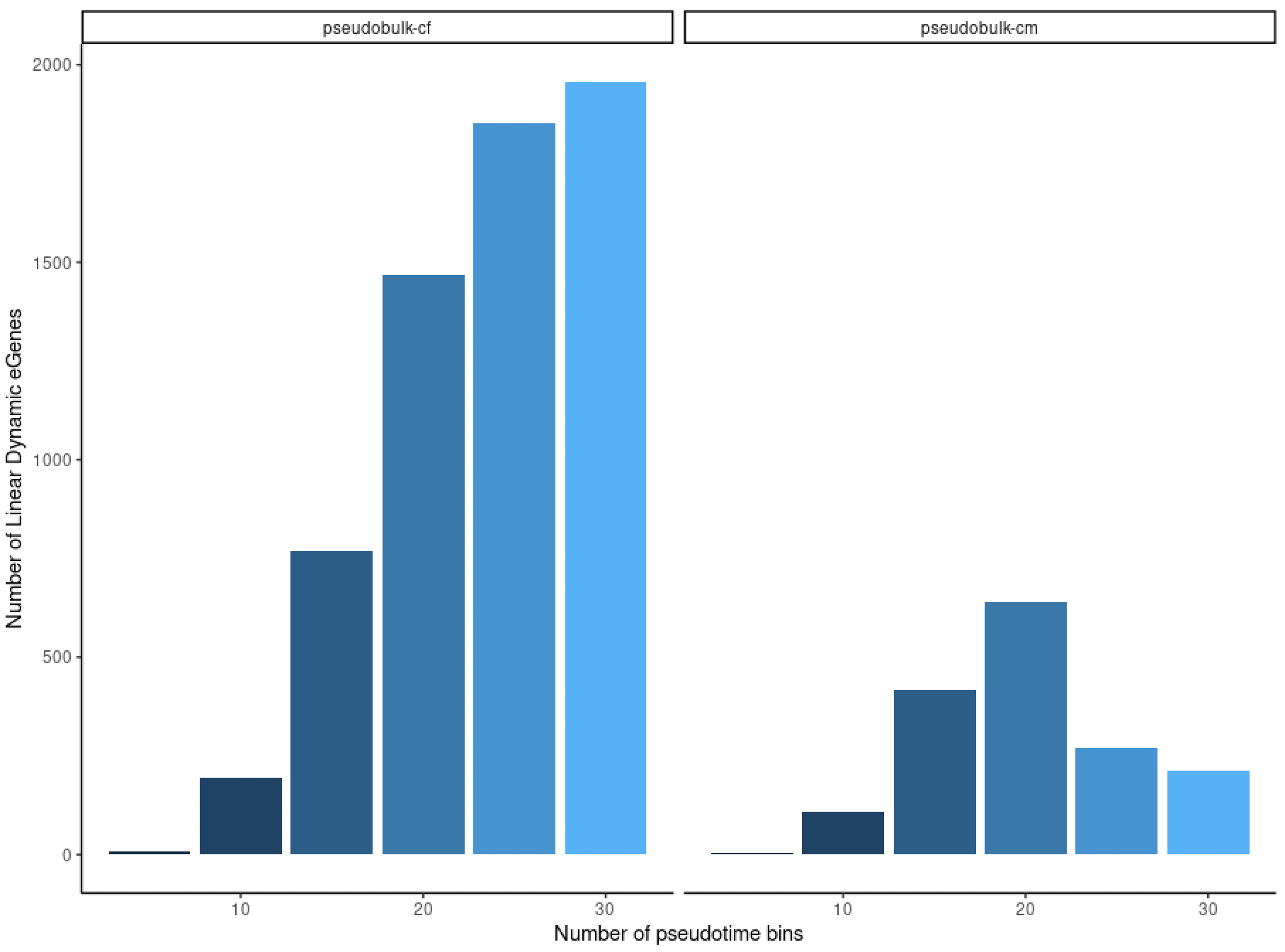

Supplement: S19 Fig — Y-axis shows the number of significant linear dynamic eGenes (genes with a dynamic eQTL, q<0.05) for a variety of numbers of pseudotime quantile bins (x-axis) for both the cardiac fibroblast (pseudobulk-cf, left) and cardiomyocyte (pseudobulk-cm, right) lineages. (TIF) [file pgen.1009666.s021.tif]

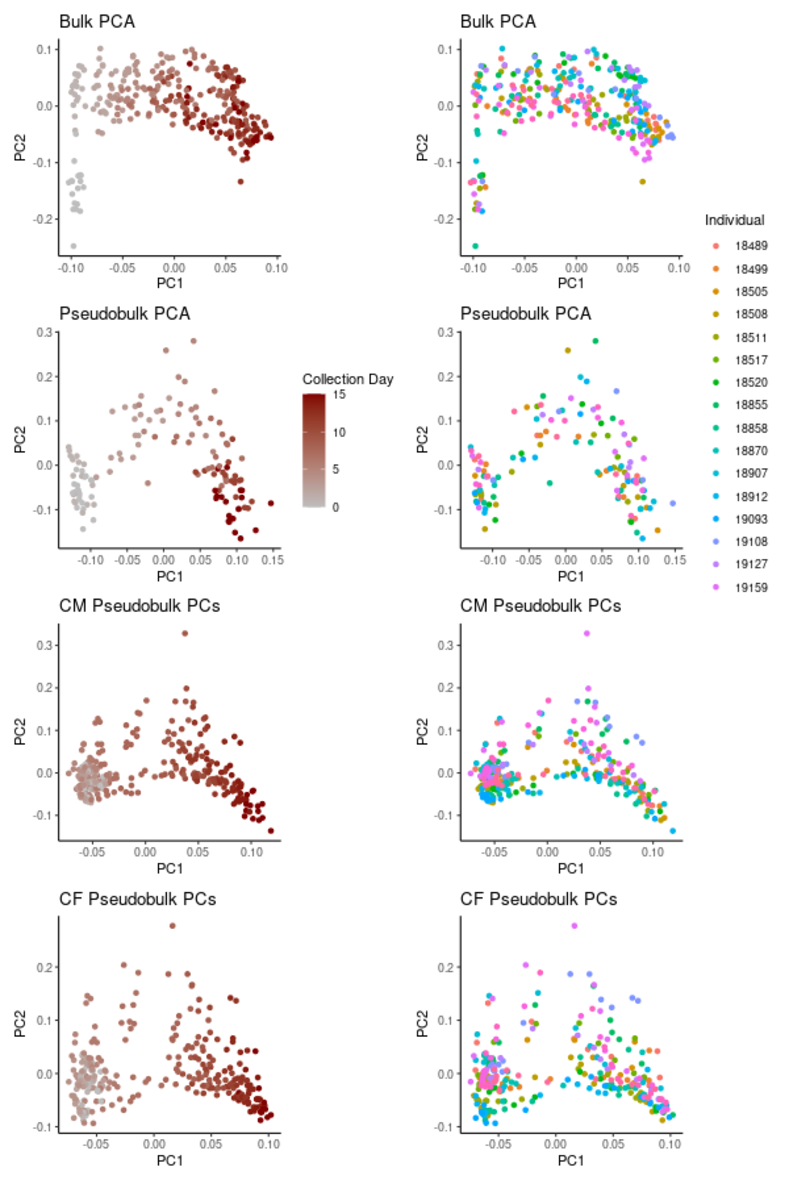

Supplement: S20 Fig — PCA on bulk (row 1), single cell data aggregated into pseudobulk by differentiation day/ individual (row 2), cardiomyocyte lineage-specific single cell data aggregated into pseudobulk by pseudotime / individual (row 3), and cardiac fibroblast lineage-specific single cell data aggregated into pseudobulk by pseudotime/individual (row 4). Samples colored on a gradient by (left column) differentiation day or pseudotime bin, or (right column) cell line. (TIF) [file pgen.1009666.s022.tif]

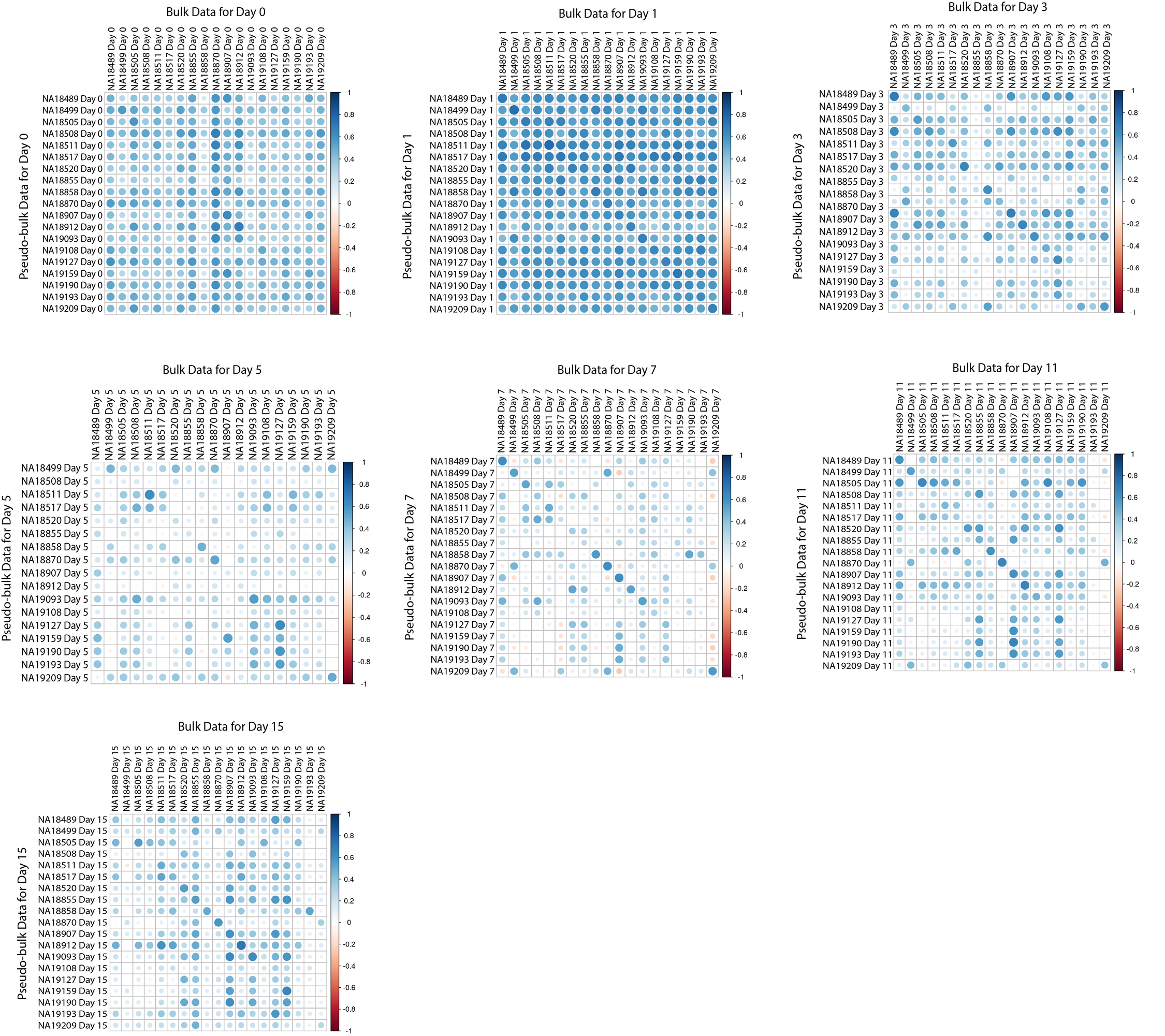

Supplement: S21 Fig — Pearson correlation between single-cell pseudobulk data and bulk RNA-seq data [23] for each individual; panels separated by differentiation day. (TIF) [file pgen.1009666.s023.tif]

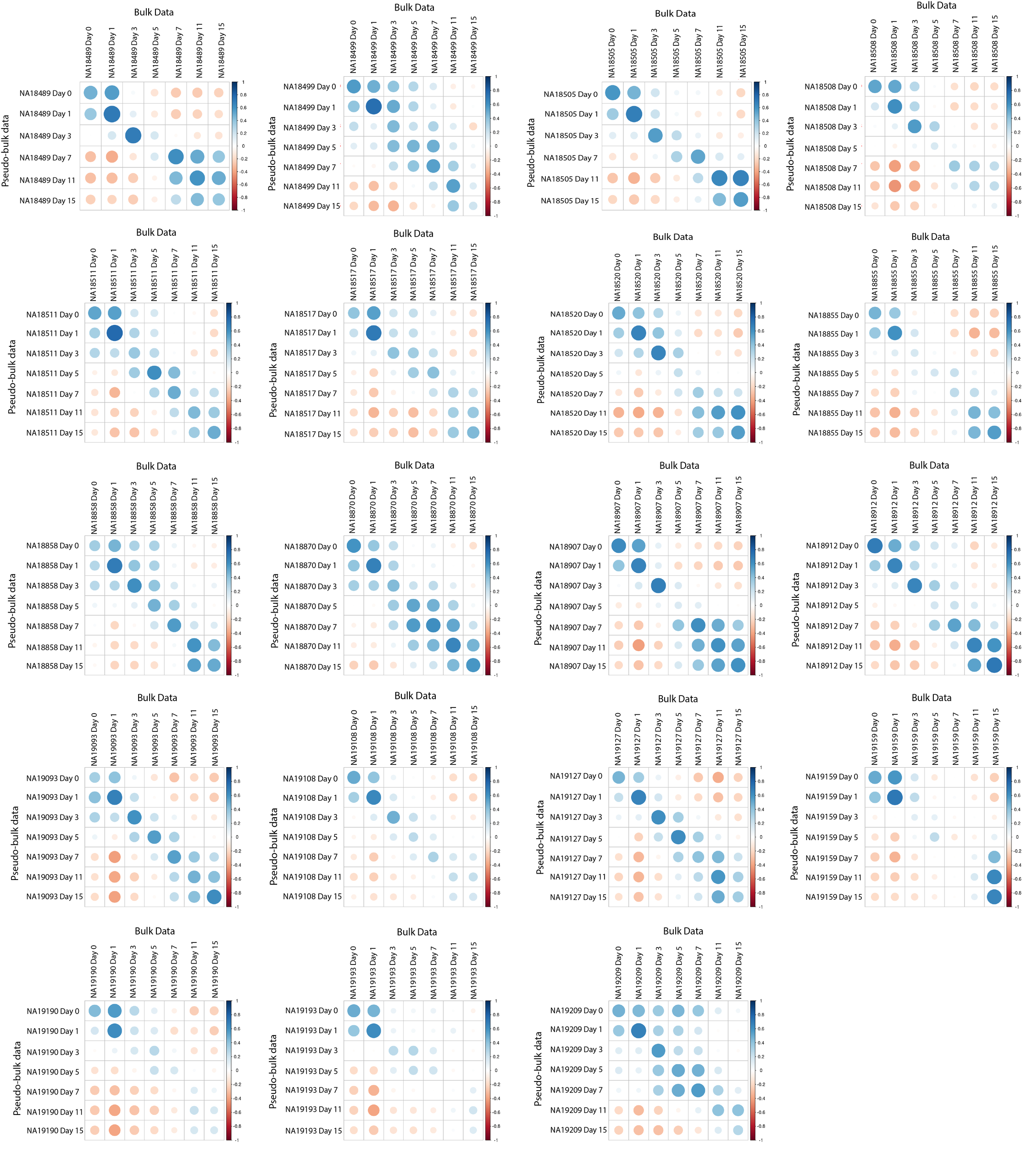

Supplement: S22 Fig — Pearson correlation between single-cell pseudobulk data and bulk RNA-seq data [23] for each differentiation day; panels separated by individual. (TIF) [file pgen.1009666.s024.tif]

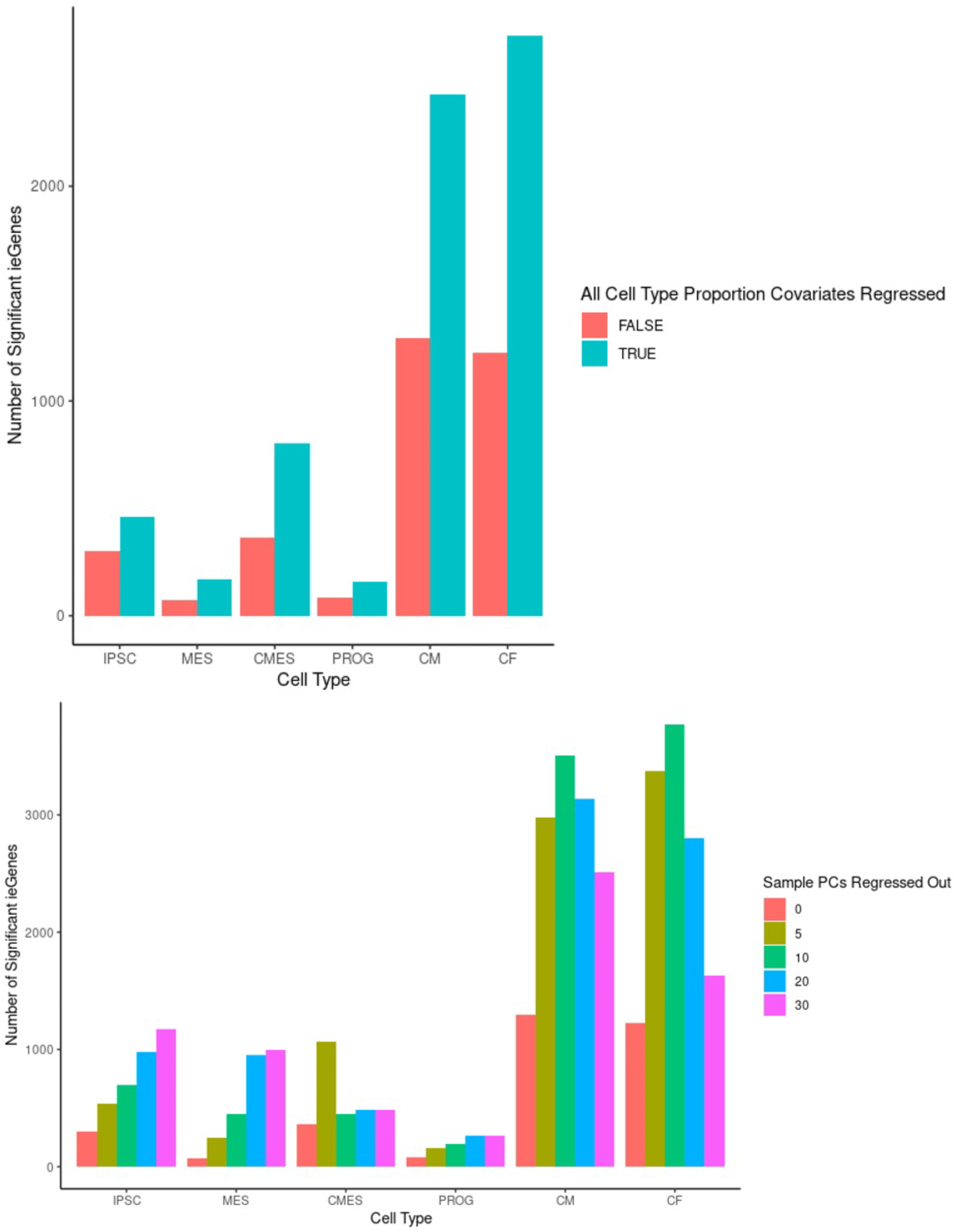

Supplement: S23 Fig — We examined the impact of regressing out additional covariates from the interaction eQTL model, and found an increase in the number of genes with a dynamic eQTL, as well as a decrease in the replication rates in bulk dynamic eQTLs (Materials and Methods) for both regression of cell type proportions (top) and up to 30 principal components (bottom). (TIF) [file pgen.1009666.s025.tif]

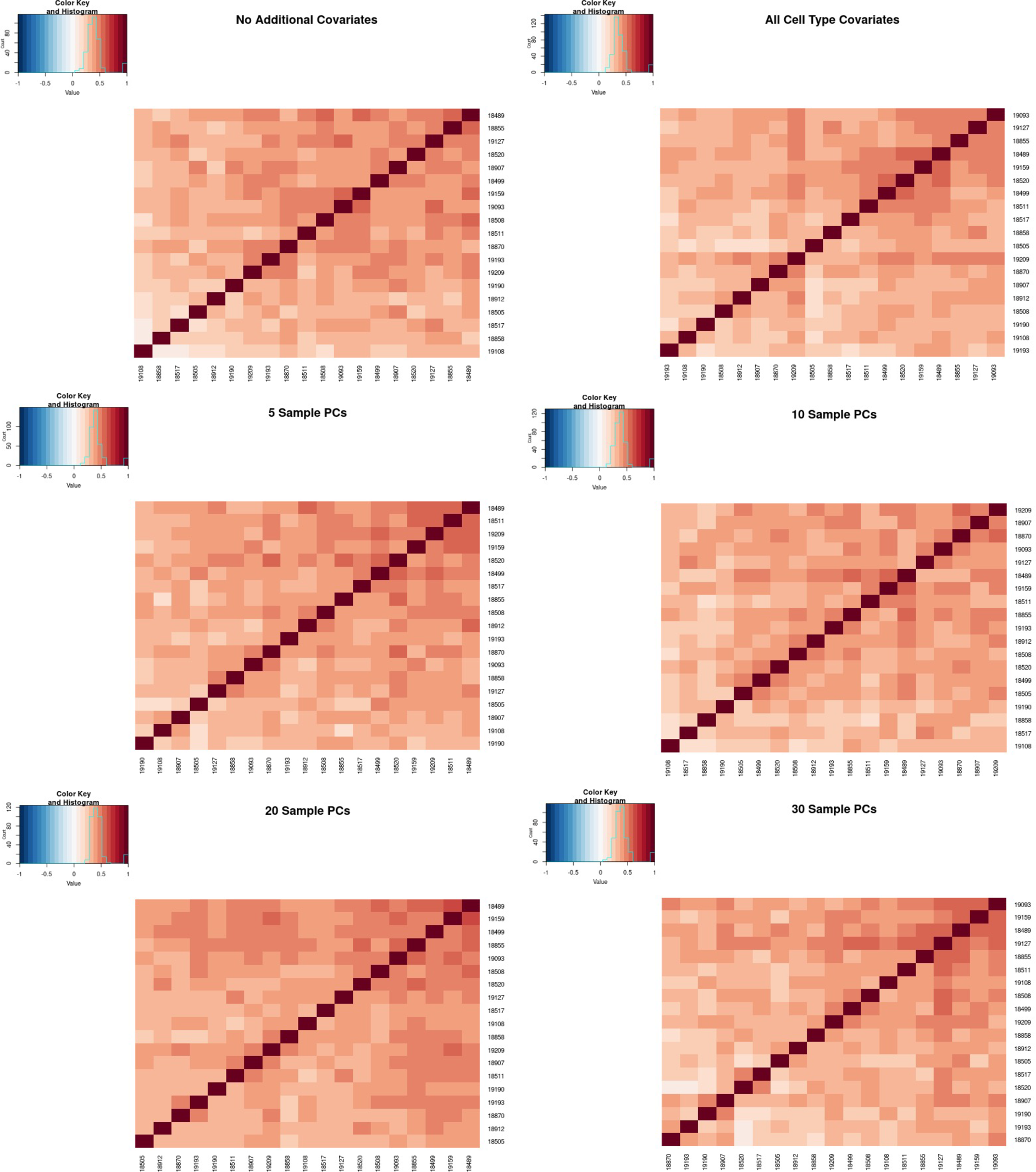

Supplement: S24 Fig — We compared genetic correlation among 200 cardiac fibroblast cell type interaction eQTLs detected exclusively after regressing out additional cell type proportion covariates (a), compared to 200 interaction eQTLs, detected before controlling for cell type proportions (b). We similarly computed genetic correlation among 200 cell type interaction eQTLs discovered only after regression of 5 (c), 10 (d), 20 (e), and 30 (f) sample principal components. (TIF) [file pgen.1009666.s026.tif]
