# Supplementary material for: Single-cell sequencing reveals lineage-specific dynamic genetic regulation of gene expression during human cardiomyocyte differentiation
Source: PLoS Genet. 2022 Jan 21;18(1):e1009666. doi: 10.1371/journal.pgen.1009666 (PMC8809621; doi:10.1371/journal.pgen.1009666)
Supplement: Attachment — Submitted filename: Response to Reviewers.pdf [file pgen.1009666.s028.pdf]

Reviewer #1

Summary

Elorbany and coauthors carried out scRNA-seq on 19 cell lines at 7 different time points and identified lineage-specific dynamic eQTLs during cardiomyocyte differentiation. The paper is easy to follow, but there are a few points that need to be addressed before this is suited to be published in PLOS Genetics.

Thank you for your summary and assessment of our paper.

Major:

1. The authors identified two major lineages, in concordance with their previous findings. The differentiation process involves transient cell states, instead of discrete cell types. I am concerned about the clustering analysis/results shown in Figure 1. A trajectory analysis will be more appropriate – granted, the authors inferred the pseudotime in Figure 2, but the results in the first figure fall in the domain of hard clustering and may raise questions. In addition to trajectory reconstruction, the authors can also consider “soft” clustering instead. For example, the PAGA method that was referenced [44].

Thank you for this comment, we agree that caution must be taken in the interpretation of the hard clustering analysis, and have added the following text to the manuscript to note this to readers (lines 181-184).

> Next, we used unsupervised clustering to partition cells into clusters, and matched cell clusters to known cell types based on expression of known marker genes (**Fig 1C**, Methods, [36]). While these discrete cell type assignments are imperfect as they fail to capture the continuity of the differentiation process, they are useful in characterizing the broad relationships between groups of cells.

We have also modified the language of the text to make clearer the role of trajectory inference in this analysis and our use of the PAGA method suggested by the reviewer (lines 239-242).

> We performed trajectory analysis using PAGA [44] to examine the relationships between the cell types present in this dataset, which helped to resolve two distinct lineages present in the data, giving rise to cardiomyocyte and cardiac fibroblast cell types (S5 Fig).

The hard clustering shown in Figure 1 as well as the bifurcating trajectories from PAGA shown in Fig S5 were the basis of lineage subsetting that was used for lineage-specific eQTL calling. Additionally, these clusters are used to generate a signature matrix for

deconvolution of bulk data in the identification of cell type interaction eQTLs. Furthermore, Figure 1 includes all cells, including cells from the unknown cell type later removed from downstream analysis, and we believe it is important to visualize the full dataset for the reader. For these reasons, we have chosen not to remove the hard clustering shown in Figure 1.

2. There are two lineages (CM and CF) identified. How is pseudotime inferred and assigned for these two lineages given there is a split? Figure 2b does not clearly show the bifurcation pattern, and the pseudotime seems to be monotonically increasing along the reduced dimensions. Did the authors carry out two lineage-specific eQTL analyses using pseudotime x Genotype as a covariate?

Pseudotime is inferred using diffusion pseudotime (Haghverdi et al. Nat Methods 2016, reference 43), which is compatible with multifurcations, so the two lineages did not have to be separated ahead of time (Methods - Lineage specification and pseudotime inference).

The bifurcation is more clearly evident in the PAGA plot (Fig S5) and the principal components biplot (Fig S2), but it is also reflected in the Force Atlas embedding (Fig 2a) by the decreased cell density observed in the region between the two terminal cell types, cardiomyocyte (CM, red) and cardiac fibroblast (CF, purple). We agree that Figure 2b did not clearly show the bifurcation pattern. We have used a new color scheme in order to more clearly show that pseudotime increases from IPSC (blue in Fig 2a) to both terminal cell types, CM and CF (red and purple in Fig 2a). We have also changed the color scheme in Fig S2 (and Fig 1a for the sake of consistency) for clarity, such that distinct colors are used for plots colored by differentiation day and those colored by cell type, so that the meaning is not confused.

We did carry out two lineage-specific eQTL analyses with cells assigned to either the cardiomyocyte lineage or the cardiac fibroblast lineage. Precursor cell types were included in both analyses. Both of these analyses measured the significance of the effect of (median pseudotime) x (genotype) on expression (this is β_{13} in line 924). The gene-variant pairs where this interaction effect was significant are designated as dynamic eQTLs. We have added the following line to the manuscript to clarify this aggregation strategy:

> Cells were assigned based on the trajectory analysis to the cardiomyocyte lineage, the cardiac fibroblast lineage, or both in the case of precursor cell types.

3. While the authors show the number of significant gene-SNP pairs in Table 1, it would be more informative to show how they compare and contrast via, for example, a venn diagram. How many of the SNPs identified by the pseudobulk are also identified by the

bulk analysis with more timepoints? How do they compare against the original bulk analysis? The authors mentioned, “This variant is not detected as a dynamic eQTL without lineage subsetting or pseudotime binning.” Is it detected by lineage subsetting (the original Science paper identified two clusters of cell lines)? In this case, the authors could more clearly emphasize the utility of the single-cell data.

Based on this suggestion, we have now included a Venn diagram of dynamic eGenes identified from the bulk and single-cell analyses, as well as the pi1 replication rates in Fig S6. While we see that a portion of cardiomyocyte dynamic eGenes and cardiac fibroblast dynamic eGenes overlap, we identify hundreds of variant-gene pairs that are unique to a single lineage. Though the bulk data was obtained from a separate experiment, a sizeable subset of the dynamic eGene-eVariant pairs are replicated in the bulk analysis (pi1=0.40 for significant eGene-eVariant pairs in the cardiomyocyte lineage, pi1=0.13 for cardiac fibroblast lineage).

Regarding the utility of the single-cell data over bulk, we re-emphasize for the reviewer that while analysis of the bulk expression data in Strober et al. supported the *existence* of two clusters of cell lines, it was not possible to deconvolve the bulk data or analyze lineage-specific dynamic eQTLs before collection of the single cell dataset presented here. Using this new single-cell resource, our manuscript outlines an approach to enable future analysis of bulk data for the identification of lineage-specific cell-type interaction eQTLs (described in the *Deconvolution of bulk RNA* section). The lineage-specific interaction eQTLs, then called in the bulk data, also provide additional support for a subset of the dynamic eQTLs called using pseudobulk data (pi1 replication rate of CM-dynamic eQTLs in CM-interaction tests is 0.41, replication rate of CF-dynamic eQTLs in CF-interaction tests is 0.14). The single cell data alone does, of course, also allow identification of additional QTLs not evident from analysis of the bulk data.

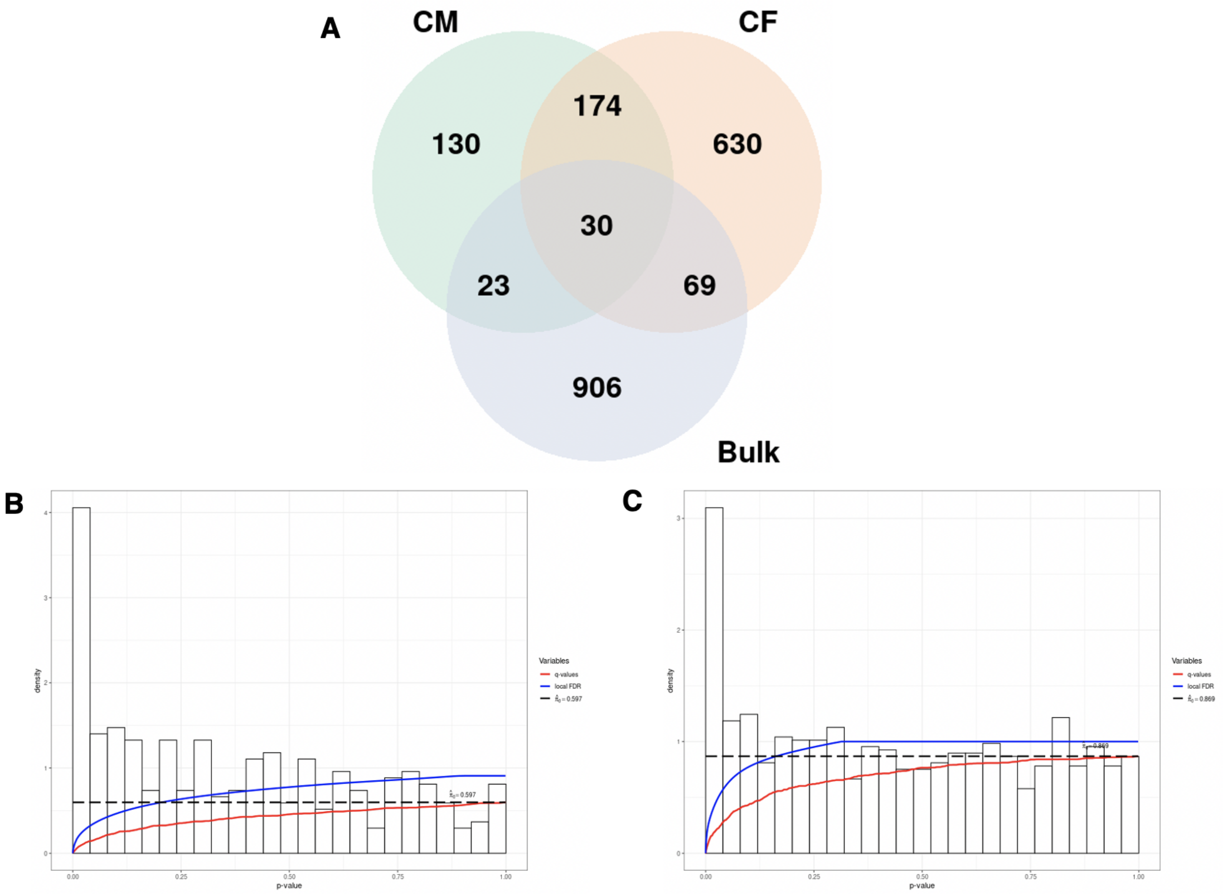

S6 Fig: **Comparison of dynamic eQTL calling in bulk and pseudobulk.** (A) Number of dynamic eGenes that were detected in common between pseudotime-binned cardiomyocyte lineage, pseudotime-binned cardiac fibroblast, and previously collected bulk data. The majority of cardiomyocyte lineage dynamic eGenes overlap with at least one of the other two analyses. (B, C) Replication analysis of pseudobulk dynamic eQTLs in bulk [77]. (B) Distribution of nominal p-values from bulk data for the subset of gene-variant pairs that were identified as a dynamic eQTL in the pseudotime-binned cardiomyocyte lineage (pi1=0.40). (C) Distribution of nominal p-values from bulk data for the subset of gene-variant pairs that were identified as a dynamic eQTL in the pseudotime-binned cardiac fibroblast lineage (pi1=0.13).

4. The authors carried out close to 2 million tests, and I am concerned about the power issue. For the two cases in Figure 3a and Figure 3c, visualization of the regression analysis is needed to show the estimated beta_13 and beta_20 with their estimates and nominal p-values (e.g., using partial residuals).

Thank you for bringing up this important point. We have added a partial regression plot and a partial residuals plot for each example dynamic eQTL in Fig S12, with estimated

effect sizes, nominal p-values, and gene-level q-values included in the caption. These plots do not suggest problems with the regression analysis (no outlier effects are observed, and we see a reasonable distribution of data points).

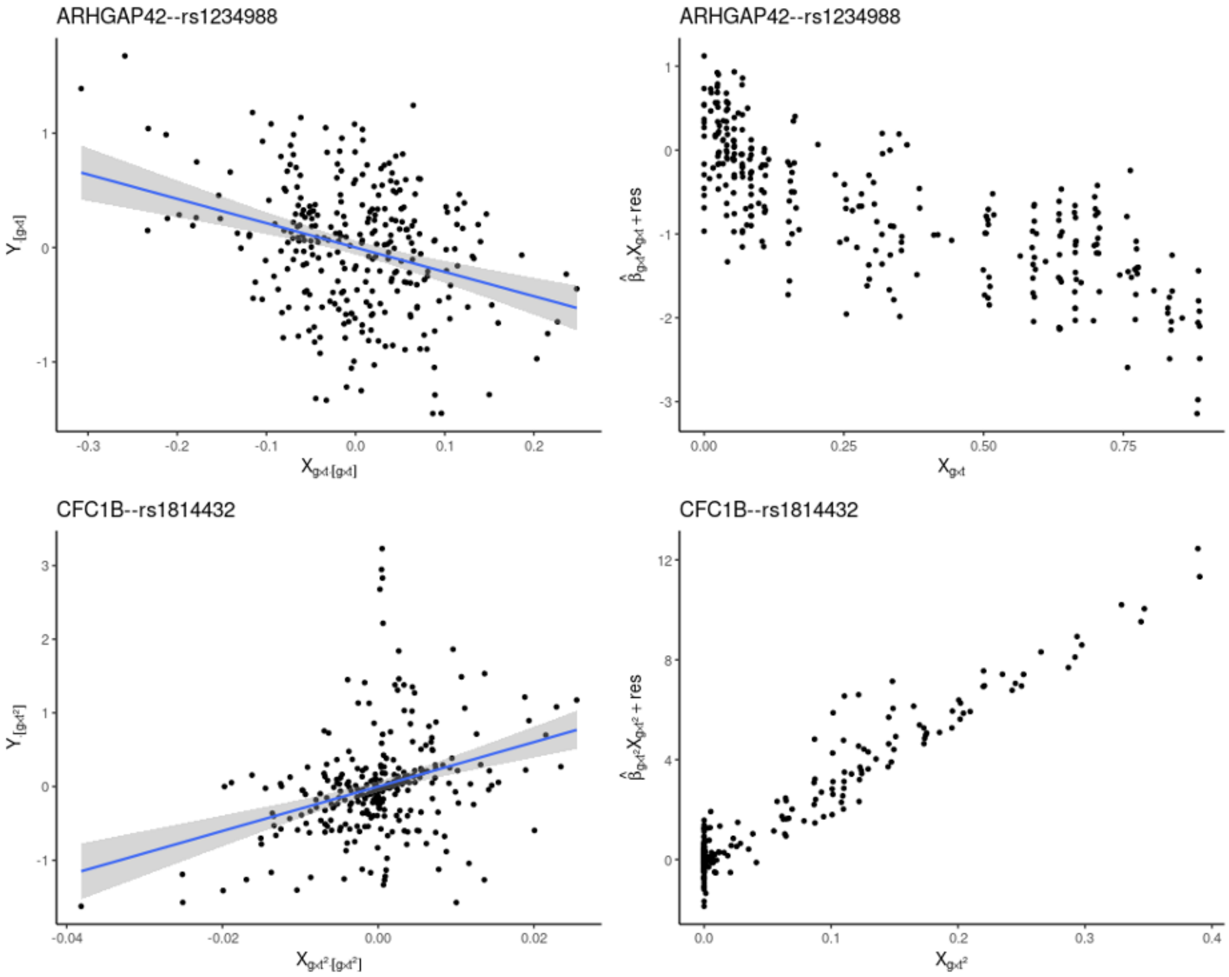

S12 Fig: Regression Analysis. (Top left) Linear dynamic eQTL, partial regression plot. For the linear dynamic eQTL example shown in Fig 3A ($\hat{\beta}_{g*t} = -2.13$, $p = 1.64 * 10^{-7}$, $q = 9.7 * 10^{-4}$), we obtained the residuals from regressing expression on all independent variables except the genotype * pseudotime interaction term (y-axis), and plotted these against the residuals from regressing the interaction term itself on all other independent variables (x-axis). The slope of the line shown measures the effect of the interaction between genotype and pseudotime after controlling for all other independent variables. (Top right) Linear dynamic eQTL, partial residuals plot. On the y-axis, $X_{g*t}\beta_{g*t} + res$, where X_{g*t} is genotype*time for a cell line/ pseudotime bin pseudobulk sample, β_{g*t} is the estimated coefficient for the genotype*time interaction term, and *res* are the residuals from the fitted linear dynamic

eQTL model. On the x-axis is X_{g*t}. (Bottom) Similar partial regression and partial residuals plots (respectively) for the nonlinear dynamic eQTL shown in Fig 3C ($\hat{\beta}_{g*t^2} = 30.1,\ p = 1.26 * 10^{-8},\ q = 1.3 * 10^{-3}$), where the interaction term of interest is between genotype and pseudotime squared.

Minor:

1. It would be good to include a reduced dimension plot with colors corresponding to batch. I understand that the authors carefully designed the experiment, but there are 131 samples – is batch effect corrected for?

We have added Fig S1 to the supplement showing the UMAP embedding colored by batch. While some cells from the same batch do appear to group closely together, the subsequent plots demonstrate that this effect stems from similarity within samples (cells from the same cell line/ differentiation day), which are likely due to biological factors, rather than similarity within batches which are more likely to be technical. Each batch contains a small number of samples, and the subsets of cells grouping together from a given batch in fact reflect samples. We have added a brief discussion of this to the *Results* section:

> While cells from the same cell line and differentiation day have similar expression profiles, likely due to biological factors, this study design minimizes technical effects associated with collection batch (**S1 Table**, **S1 Fig**).

Furthermore, we show in the analysis described below that cell type composition is not impacted by batch. Therefore, batch is not directly regressed out as this would remove some of the variation due to real biological effects relating to cell line and differentiation day. (Batch is defined as the experiment, collection day, and collection in which cells were collected. The experimental design is now described in Table S1).

> ***Influence of batch on cell type composition*** If a group of cells from the same batch are more likely to display similar cell type composition than cells from different batches, this could indicate that batch effects are confounding cell type annotation. However, since the experimental design places cells from the same cell line and differentiation stage in the same batch, it is important to disentangle similarity in cells due to the same cell line/ differentiation stage (a likely biological effect) from similarity in cells due to batch (a primarily technical effect; see S1 Fig). To do this, we grouped cells by cell line and differentiation day (called a ‘sample’ here), and summarized each group by their cell type proportions. As indicated in **S1 Table**, each batch contains three different samples representing a different cell line/ differentiation day combination. We focused on the impact of batch on terminal cell type composition by analyzing samples from collection

day 3 that had been differentiating for 11 or 15 days. At these time points, terminal cell types (cardiomyocyte, CM, and cardiac fibroblast, CF) make up a majority of the cells present. Based on the experimental design, we have 19 pairs of samples such that one sample is at differentiation day 11, the other is at day 15, and the two samples belong to the same batch. If batch effects are causing cells from the same batch to receive similar terminal cell type labels, we would expect these sample pairs to display greater similarity of cell type proportion compared to a background of sample pairs from distinct batches matched for differentiation day. We computed pairwise differences in cell type proportion for both cardiomyocyte and cardiac fibroblast cell types, and compared these to a background (day 11 and day 15 samples from distinct batches) using a t-test. This analysis indicated that samples from the same batch are not more likely to display similar cell type composition than cells from different batches when controlling for cell line and differentiation day (CM $p=0.286$, CF $p=0.446$).

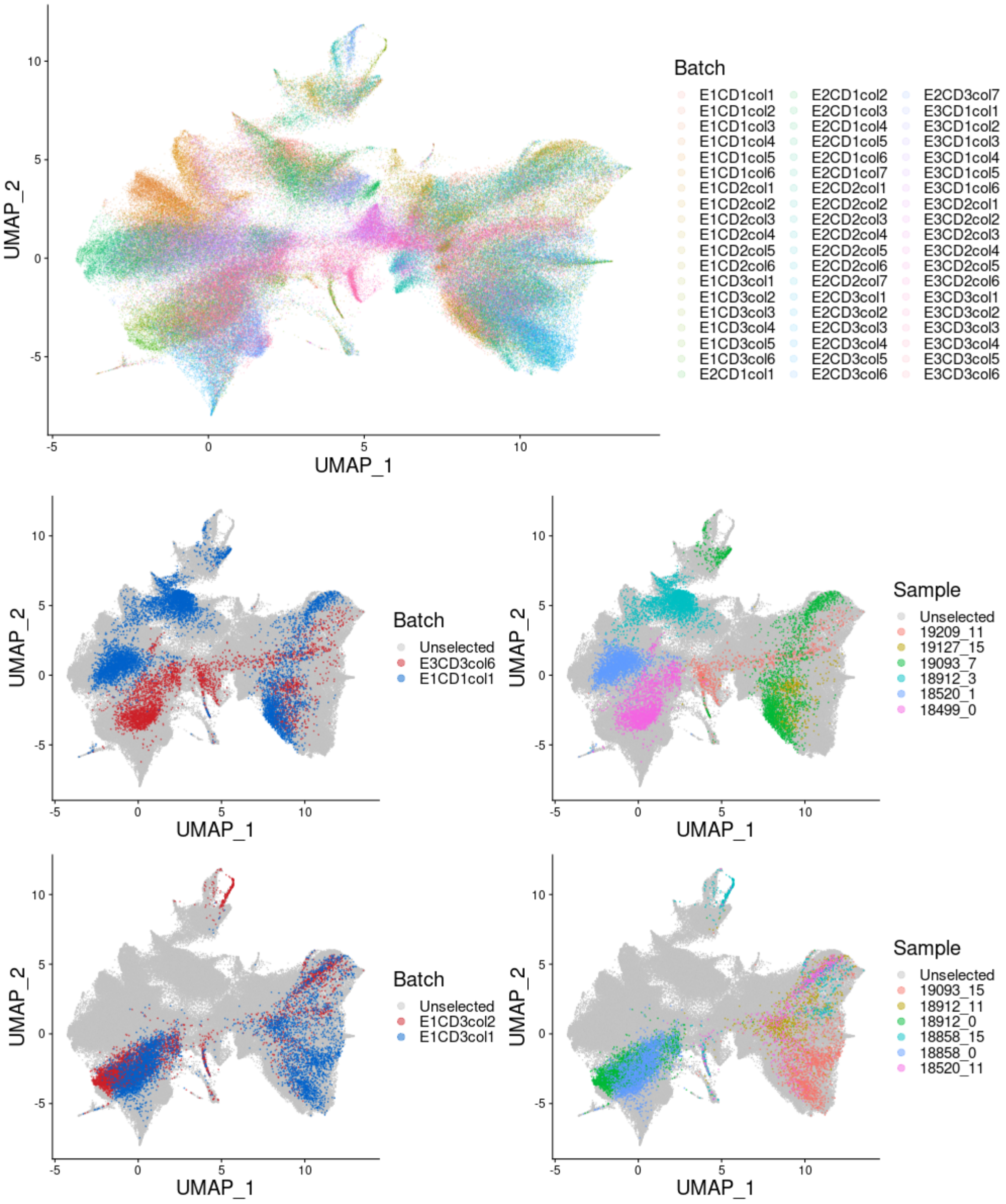

S1 Fig: UMAP embedding colored by batch and sample. (A) Cells are colored by batch (the experiment, collection day, and collection in which they were collected for sequencing). (B-E) Coloring by batch (B, D) and sample (C, E) shows that apparent batch effects are driven by similarity between cells of the same sample (cell line and differentiation day) within the batch, rather than the overall batch itself.

2. It would be more appropriate to reduce the dimensions and mark the two lineages after removing the unknown cells, as was done in Figure 2.

We have added supplementary figure S18, which shows a UMAP embedding of the data after removing the unknown cells, and reference this in the *Lineage specification*

and pseudotime inference subsection of the *Methods*. Since the unknown cell type was included in the deconvolution analysis later in the paper (and did account for a substantial proportion of the cells in a few bulk samples), we believe it is appropriate to include them in Figure 1 to avoid confusion for the reader and ensure the full dataset is represented initially. The two lineages are first treated as distinct in Figure 2 (C-F), after the unknown cells have been removed.

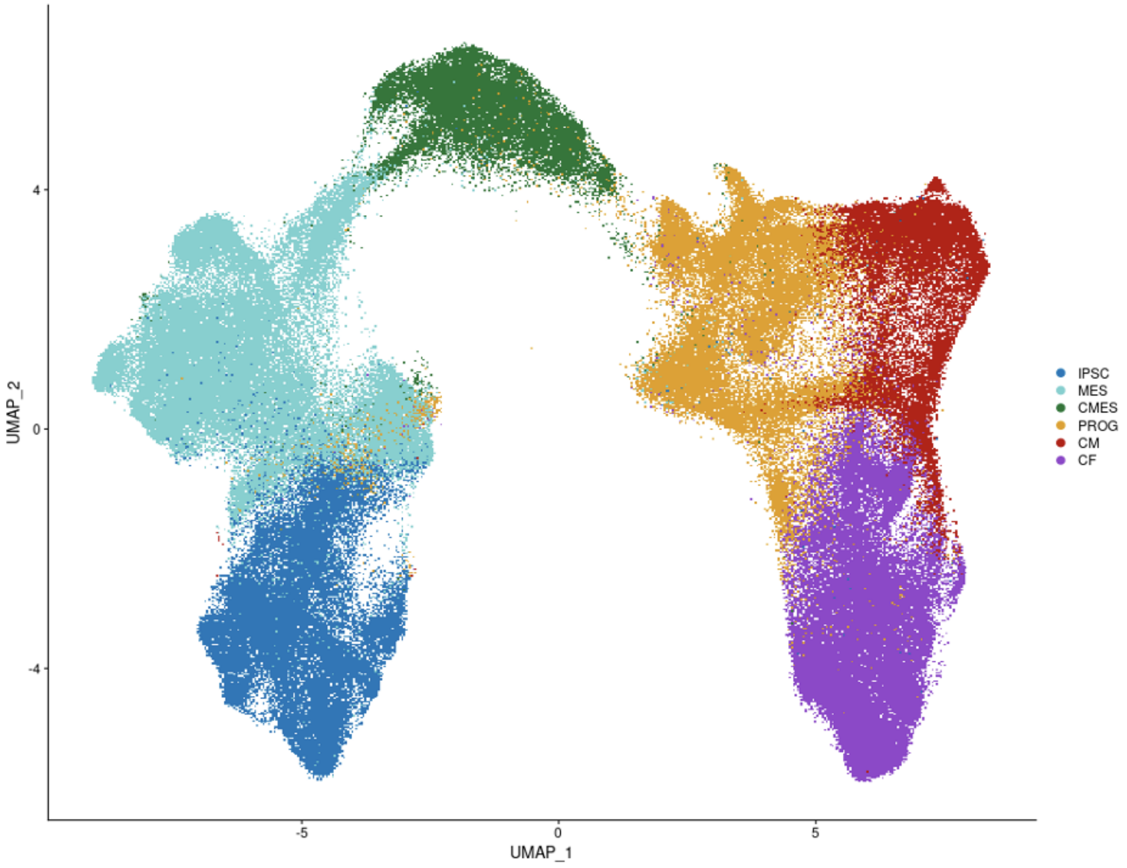

S18 Fig: UMAP embedding with outlier clusters removed. As in Fig 1C, a UMAP embedding of the single cell dataset colored by cell type, except with outlier clusters removed.

3. The figure legends and axis labels are too small to be read.

Thank you for this recommendation, we have increased the font size in the legends and axis labels for figures 1, 2, and 3.

4. In Figure 3A, boxes for GG at timepoint 14 and 15 are missing.

There is no data at these timepoints for genotype GG because these timepoints from the single individual with genotype GG were removed based on their library size (these samples had less than 100,000 total UMIs). We have described this thresholding in further detail in the *Pseudobulk expression aggregation and normalization* section of the *Methods*, and added the median number of samples from each genotype to the legend of Figure 3 to further clarify and address a comment from Reviewer 2.

Reviewer #2

Summary

The authors perform single cell analysis of 19 donors across 7 time points in order to examine dynamic genetic regulation during differentiation of cardiomyocytes. They find both linear and non-linear dynamic eQTLs, a number of which would not have been found without the use of single cell resolution, which enabled pseudotime reconstruction and lineage subsetting. The disentangling of cell line specific differentiation speed is a key result, and the detailed analysis presented here provides a framework for future studies. The authors then compare their results to a previous bulk eQTL time series dataset and to GTEx. Finally, the authors use cell type proportions per cell line to deconvolve the bulk samples and detect cell type interaction eQTLs. The dataset and analysis presented here are both valuable for increasing the understanding of dynamic genetic regulation of these cell types, as well as for the discussion of technical issues and optimizations that should be taken into account when performing single-cell QTL studies from iPSC.

Thank you for the summary and comments on the value of the manuscript.

Major comments:

- "We collected single-cell data using a balanced study design in which each collection included three individuals at three unique differentiation time points." Please provide the study design as a Supp Table or additional data table. Do the collection groups from the study design correlate with the two clusters of cell lines with respect to the bifurcation in cell fate?

We have added the study design as Table S1. To address the second point, whether batch effects are driving the cell-line level differences in cell type composition, we have added the following analysis to the supplement, which suggests that batch effects are not responsible for these differences in cell type composition:

> ***Influence of batch on cell type composition*** If a group of cells from the same batch are more likely to display similar cell type composition than cells from different batches, this could indicate that batch effects are confounding cell type annotation. However, since the

experimental design places cells from the same cell line and differentiation stage in the same batch, it is important to disentangle similarity in cells due to the same cell line/ differentiation stage (a likely biological effect) from similarity in cells due to batch (a primarily technical effect; see S1 Fig). To do this, we grouped cells by cell line and differentiation day (called a 'sample' here), and summarized each group by their cell type proportions. As indicated in **S1 Table**, each batch contains three different samples representing a different cell line/ differentiation day combination. We focused on the impact of batch on terminal cell type composition by analyzing samples from collection day 3 that had been differentiating for 11 or 15 days. At these time points, terminal cell types (cardiomyocyte, CM, and cardiac fibroblast, CF) make up a majority of the cells present. Based on the experimental design, we have 19 pairs of samples such that one sample is at differentiation day 11, the other is at day 15, and the two samples belong to the same batch. If batch effects are causing cells from the same batch to receive similar terminal cell type labels, we would expect these sample pairs to display greater similarity of cell type proportion compared to a background of sample pairs from distinct batches matched for differentiation day. We computed pairwise differences in cell type proportion for both cardiomyocyte and cardiac fibroblast cell types, and compared these to a background (day 11 and day 15 samples from distinct batches) using a t-test. This analysis indicated that samples from the same batch are not more likely to display similar cell type composition than cells from different batches when controlling for cell line and differentiation day (CM p=0.286, CF p=0.446).

- A number of results are not presented in Results in the order I would have expected. The simulation to assess double dipping from latent variables in the linear model formula (S18) doesn't follow directly the section on the linear model but shows up in the Discussion. Likewise the permutation analysis (S10) is only mentioned in Methods. These seem to be key control experiments that should be at the least briefly mentioned in main text.

Thank you for this suggestion to improve the flow of the paper. We have moved a revised version of the discussion of double dipping from latent variables into the *Mapping of dynamic eQTLs* section, as well as a description of the other control experiments. We have also renamed the *Permutation Analysis* section of the *Methods* to *Control Experiments*, where several control experiments are described in greater detail.

Minor comments:

In line 209, it is described how and why single cells were aggregated within pseudotime bins separately along each lineage. This facilitates analysis, allows for use of

well-developed tools for bulk profiles, and as described in the text makes analysis perhaps more robust. Can the authors discuss any potential downsides to this approach compared to a model at the single cell level using the continuous inferred pseudotime variable?

Thank you for bringing up this important point. Downsides of pseudobulk aggregation include that it constrains us to exploring lineages that are defined with relatively low pseudotime resolution compared to the full continuum of possible pseudotime values. This could limit the space of hypotheses we are able to explore describing the relationship between gene expression and pseudotime, such as changes that occur within one of our bins. We ultimately decided these factors did not outweigh the computational burden and high false positive rates that have previously been reported to occur with direct eQTL calling on single-cell data (Cuomo et. al. *bioRxiv*. 2021, Reviewer Response Ref. 1), particularly at our sample size. However, we appreciate that this point deserved more discussion in the manuscript. Therefore, we now make further mention of the tradeoff between aggregation and single-cell analysis in the section *Single-cell expression data resolves bifurcating trajectories during cellular differentiation*.

From line 221, the dynamic eQTLs are presented, e.g. 357 eGenes in CM and 903 in CF. These are the significant results from interaction of genotype and differentiation time. How many stable eGenes were found? That is the significance of beta_1 in line 703. Or are stable, genetically regulated eGenes not possible to detect with their analysis framework?

The coefficient beta_1 is an estimate of the effect of the non-reference allele on expression, *specifically at t=0*, where t is median sample pseudotime or collection time, depending on the model being considered. This is not equivalent to the stable or shared effect across time, so the significance of beta_1 in our joint model does not directly allow us to identify stable eGenes.

However, we performed additional analysis in response to this reviewer question. One way to identify eQTLs that are active across time points or contexts is to perform eQTL calling in each context independently, and then pool information to infer patterns of sharing of regulatory effects across contexts, as done by the *mashr* package (Urbut et al. *Nat Gen* 2019, reference 73). We have added a section to the supplement called *Static eQTL calling* describing this analysis, with the key result added to the *Mapping of Dynamic eQTLs* section. In this way, we found 183 static eQTLs using pseudobulk data aggregated by differentiation day, versus 147 static eQTLs using pseudobulk data aggregated by cell type.

"Ultimately, our lineage subsetting and pseudotime approach revealed more dynamic eQTLs than were previously identified...The increased detection rate may stem from...", this is technically true but the 3% increase might be better described as "nearly the same" or "slightly more". At first I missed that this was the increase being described.

We have modified this language in this section to make the comparisons more clear. We describe the mentioned increase as “slightly more”. We have also added the following to lines 294-296 in order to highlight that when the bulk data is subset to the same smaller number of collection time points as were used for the single cell experiment, substantially fewer dynamic eQTLs are identified than in the single cell analysis:

> When the bulk data was subset to the same 7 collection time points used for the single cell experiment, only 210 dynamic eGenes were detected.

In Fig 3a and 3c, it would be useful to know the number of samples per genotype x timepoint, if there is a condensed way to include this information on the plot. At the least, knowing the mean number of samples per genotype would be useful.

We have described this subsetting process in further detail in the *Pseudobulk expression aggregation and normalization* section of the *Methods*, and added the median number of samples from each genotype to the legend of Figure 3.

Line 266: recommend to cite qvalue here to give background to the pi_1 statistic.

We have added a citation to the qvalue paper as suggested.

Line 270 "by searching directly for dynamic effects across tissues rather than within a single tissue in isolation": Suggest rewording "across cell types" rather than "across tissues" as in principle the analysis presented does not require a multi-tissue system.

Thank you, the language in this section has been modified as suggested.

Line 298: any hypothesis as to why the CF replication was much lower than the CM replication as estimated by pi_1?

This is an interesting question. One explanation may be that cell composition and differentiation time are related differently in the CM lineage from the CF lineage. Specifically, while we generally expect cellular composition (the independent variable of interaction eQTL analysis) to be related to differentiation time (the independent variable of dynamic eQTL analysis), the correlation between the two varies between lineages (Pearson’s ρ=0.59 for cardiomyocyte proportion and differentiation day, compared to ρ=0.36 for cardiac fibroblast proportion). This may reflect a fundamental difference between the two differentiation trajectories, for example, if samples which produce primarily cardiac fibroblasts reach maturity more quickly. This hypothesis is supported

by the observation that the maximum pseudotime value of the cardiomyocyte lineage is greater than that for the cardiac fibroblast lineage. There may be other properties contributing to replication differences, but this is one likely factor. We have added this consideration to the manuscript:

> Interaction eQTLs for a cell type at an endpoint of the differentiation (iPSC, cardiomyocyte [CM], and cardiac fibroblast [CF]) are expected to be related to linear dynamic eQTLs, since cellular composition often partially reflects differentiation time. However, this relationship varies between lineages: we find that cardiomyocyte proportion is more correlated with differentiation day than cardiac fibroblast proportion (Pearson's ρ=0.59 for cardiomyocyte proportion, compared to ρ=0.36 for cardiac fibroblast proportion). Accordingly, we found that CM and CF ieQTLs called with this approach were replicated in the previously used dynamic eQTL calling framework on the same bulk dataset to a varying degree (pi1=0.84 and 0.43, respectively). This may reflect a fundamental difference between the two differentiation trajectories, if cells within samples which produce primarily cardiac fibroblasts reach maturity more quickly. This hypothesis is supported by the observation that the maximum pseudotime value of the cardiomyocyte lineage is greater than that for the cardiac fibroblast lineage (Fig 2B).

Line 301: how many of the ieQTLs were lineage specific?

Of the 1295 cardiomyocyte ieGenes and 1222 cardiac fibroblast ieGenes, 78 and 77% were lineage-specific, respectively (this number has been added to the manuscript in line 410).

Line 385: "infeasible": for the sake of specificity, do the authors mean that latent variable inference is computationally intensive and thus would be a bottleneck with current algorithms?

Yes, we were referring to the computational cost of performing pseudotime inference hundreds of times or more, as would be required for the Jackstraw procedure. We have modified the language of this section (now briefer since most of the discussion of double dipping has been moved to *Mapping of dynamic eQTLs*) to frame this issue as a bottleneck rather than making the claim that it is infeasible.

Line 389-391: the fixed-effect linear model may nevertheless lead to loss in sensitivity as it is quite a bit overly conservative (as mentioned in Methods).

Thank you for pointing this out, we have modified this sentence to acknowledge the potential loss of sensitivity demonstrated by these simulations:

We demonstrate in simulation that the fixed-effect linear model used in this study was conservative in the presence of multiple measurements per individual and did not lead to type I error inflation, though there may be some loss in sensitivity (Methods, **S10 Fig**).

Line 661: "Each cell line has a shared loading across all time points, and PCs reflect trajectories across all genes" I followed the cell line collapsed PCA up until this point. Rows represent cell lines and columns represent gene x time points. So the loadings for each PC are of dimension genes x time points (~38k x 7)? I wasn't clear how this reflects trajectories.

Briefly, to clarify terminology, we are using the term loadings to describe the loadings of each PC on a cell line, such that performing cell line PCA on a (19 x (38k x 7)) matrix produces a loadings matrix of dimension (19 x k), where k is the number of cell line PCs, and a factor matrix of dimension (k x (38k x 7)). However, with regard to your point, it is true that each column of the factor matrix is of dimension genes x time points (7 for pseudobulk aggregated by day, 16 for pseudobulk aggregated by pseudotime bin). We have added further explanation of the claim that cell line PCs reflect trajectories, as well as a supporting supplementary figure, that will hopefully emphasize this point.

Each cell line has a shared loading across all time points, and PCs reflect broad differences in the way cell lines proceed through differentiation. For example, in bulk, it appears that the first cell line PC picks up on differences in differentiation speed between cell lines, while the second cell line PC picks up on differences in terminal cell type preference as defined as the highest total cell type proportion among days 10 to 15 (S9 Fig).

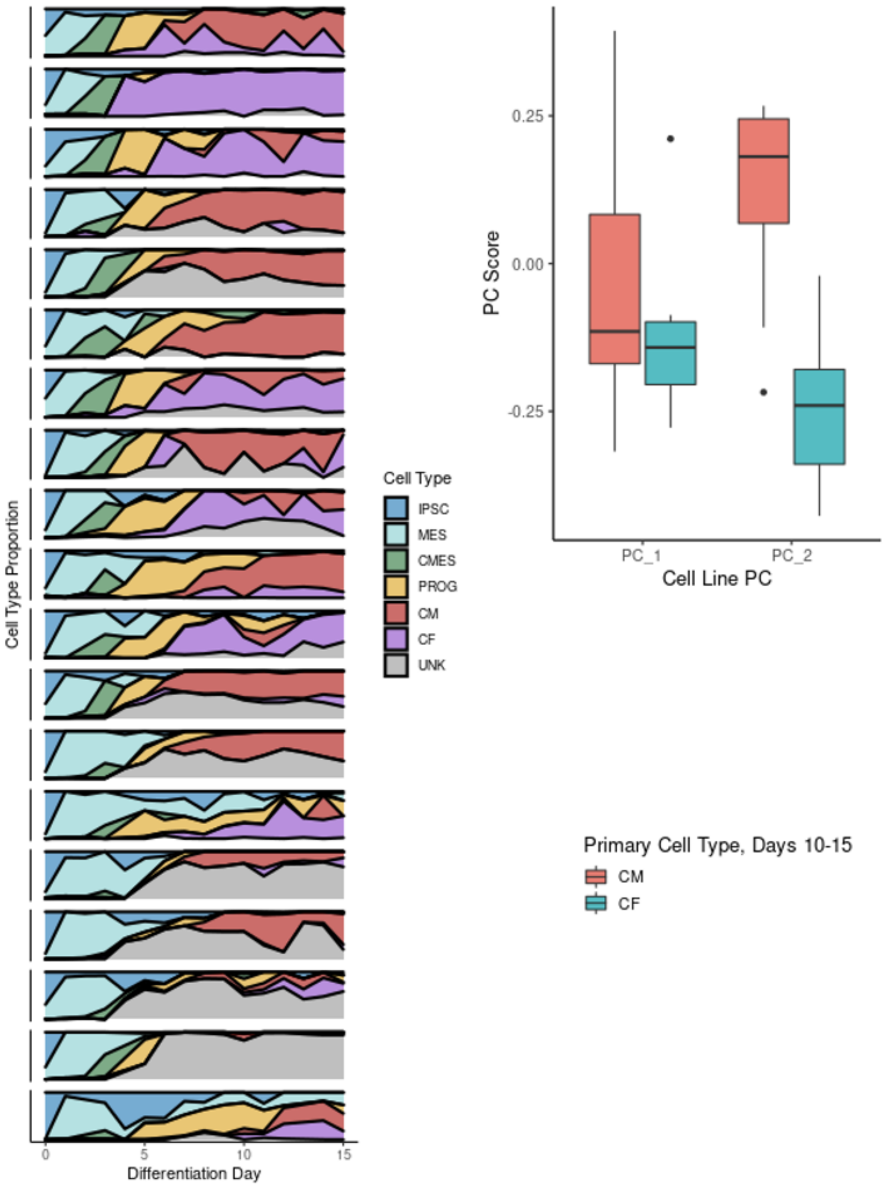

S9 Fig: Cell line PCA picks up on differences in the way cell lines progress through differentiation. (Left) Inferred cell type proportions in bulk for each of the 19 cell lines, sorted by cell line PC1 loading. Focusing particularly on the proportions of iPSC and mesoderm cells, (blue and teal, respectively), it appears that cell line PC1 is picking up on differentiation speed, with cell lines with a higher PC1 score (lower subplots) differentiating slower than cells with a lower PC1 score. (Right) The second cell line PC score appears to separate cell lines based on their terminal cell type preference, cardiomyocyte or cardiac fibroblast, as defined by the most common cell type among differentiation days 10 to 15.

Line 732: "we checked whether dynamic eQTLs were enriched for genotypes shared between any particular pair of individuals (suggesting broad individual differences could be driving the dynamic eQTLs, Fig S10)." Didn't understand what it meant for "eQTLs to be enriched for genotypes", can the authors be more specific? And Fig S10 is the permutation analysis only, was there another Fig to be referenced here (S15)?

Thank you for pointing out the figure reference error, we have changed the figure reference from Fig S10 to Fig S15. We also thank the reviewer for pointing out this confusing language, and we have replaced this line with a more thorough explanation of this analysis in the *Control Experiments* (previously *Permutation Analysis*) section of the *Methods* as quoted below:

> As another check for confounding factors, we explored the possibility that broad differences between cell lines, such as variation in differentiation speed or trajectory preference, are driving false positive discoveries. If a pair of cell lines share properties such as trajectory preference that confound eQTL analysis, variants where those cell lines share genotype would be more likely to appear as dynamic eQTLs. If this is the case, we would expect pairwise correlation between cell lines according to genotype, across the top 200 dynamic eQTLs, to be higher than expected by chance (compared to a background set of random loci matched for minor allele frequency and distance to transcription start site), as these false dynamic eQTLs are not in fact picking up on distinct *cis* regulatory patterns but broad cell line patterns. This type of elevated correlation was not observed, suggesting that cell line PCA adequately controls for broad cell line differences (**S8 Fig**).

We have additionally added a reference to this analysis in the *Mapping of Dynamic eQTLs* section alongside the other control experiments, and swapped two of the subplots in Fig S15 which had previously been placed out of order (‘CM, 16 Bins, Dynamic eQTL Variants’ and ‘CM, 16 Bins, Background Variants’).

Line 767 - extra line break.

Thank you, the line break has been removed.

Fig S10 "do not suggest inflation", would suggest "do not suggest substantial inflation" as there is some inflation of small p-values for (a) and (c) panels here.

Thank you for pointing this out, we have modified the language here to “do not suggest substantial inflation”.

Reviewer 3

Comments:
Overall, the manuscript is well written, and information is presented in a logical manner

Thank you for this comment on our manuscript.

Around line 272, the authors comment on the overlap between linear/nonlinear eQTLs identified in the cardiomyocyte lineage with eQTLs identified across all tissues in GTEx. For both the linear and nonlinear eQTLs, this overlap is ~30%. The authors mention (line 260) that nonlinear QTLs can transiently alter expression during differentiation, and thus genetically determined expression differences may not be evident in the terminal cell types. As such, I would anticipate that nonlinear QTLs would be less likely to replicate than the linear QTLs with GTEx. The manuscript could be strengthened by adding a couple sentences in this section discussing the implications of the similar replication rate between the linear / nonlinear eQTLs and why this was observed.

Thank you for pointing this out. Transient effects are actually only a portion of the nonlinear dynamic eQTLs that we detect. For example, there can be quadratic effects where the strongest regulatory effect is actually at the end of the time series in a terminal cell type, which we would not be surprised to see replicate in GTEx. We have edited our introduction of nonlinear dynamic eQTLs to make it clear not all nonlinear eQTLs are transient:

> We therefore used these values to also identify nonlinear dynamic eQTLs, whose effects vary in a nonlinear way over the course of differentiation, such as presence only at intermediate stages of the differentiation (**Fig 3C,** S12 Fig).

Further, we have now included a breakdown of the nonlinear dynamic eQTLs into “early-acting”, “late-acting”, “transient”, and “switch”. Most are classified as late-acting, which supports the high rate of replication in GTEx. None of the five early or transient cardiomyocyte nonlinear dynamic eQTLs that were discovered are present in GTEx. We have added the following section of the manuscript to highlight this point (italics indicate added text):

> … Similarly, only 22 of 75 (29.3%) nonlinear dynamic eQTLs on the cardiomyocyte lineage were previously identified as eQTLs in GTEx. *Further classification of cardiomyocyte lineage nonlinear dynamic eQTLs as “early-acting”, “late-acting”, “transient” (or "middle"), and “switch” (as in ref. [23]) found that most of these variants (68 of 74) are late-acting, supporting the similar replication rates between linear and*

nonlinear dynamic eQTLs. Of the five early-acting and transient dynamic eQTLs, none were identified as cis-eQTLs in GTEx.

In the "Mapping of dynamic eQTLs" section, the manuscript could be strengthened by commenting on why there are many fewer dynamic eQTLs for the cardiomyocyte lineage compared to the fibroblasts.

- Is there a large difference in the number of cells captured in this lineage, and so perhaps this is due to a discrepancy in power?
- If not due to discrepancy in power, perhaps it reflects that disruptions in expression over fibroblast trajectory are more deleterious to organismal fitness?

Thank you for raising this question. We agree the number of cells is an important factor, and we have added the following explanation to the section *Mapping of dynamic eQTLs:*

> The difference in the number of dynamic eQTLs detected between the two lineages may arise due to greater heterogeneity among predominantly cardiomyocyte samples, or from a difference in the number of cells captured (45,980 cardiac fibroblasts versus 21,862 cardiomyocytes) leading to more precise pseudobulk expression profiles in predominantly cardiac fibroblast samples.

While a difference between the two trajectories in the impact of variation on fitness is still possible, we do not have direct evidence to support this here.

As constructed, the cell type interacting QTLs analyses likely identify QTLs that largely overlap with the pseudotime-based QTLs identified in the previous section, as the proportion of the terminal cell type proportions are highly correlated with their respective pseudotimes. Given this, and given that the cell type interacting QTLs section mainly focuses on the results of the two terminal cell types, this section does not add much to the manuscript. To improve the contribution of this section to the overall manuscript story, one suggestion I have would be to analyze and discuss the genetic determinants of the ratio of the proportions of the terminal cell types (i.e. genetically determined lineage preference).

Thank you for the opportunity to clarify the benefits of this analysis. The key point of this section is demonstrating the utility of single cell data as a supplemental source of information that can be used to help analyze current and future bulk data to gain insights into context specificity that would be otherwise obscured by bulk aggregation across multiple cell types. While the specific interacting QTLs presented here overlap with our pseudotime-based eQTLs, the framework demonstrates the added value that our single-cell data provide to both existing and future bulk datasets. This paradigm for analysis is potentially powerful, for example, given bulk data with larger sample sizes or denser time point sampling possible due to lower cost and effort of bulk sequencing,

combined with smaller-scale single cell data that offers cell type, lineage, and pseudotime resolution. In fact, given the denser time-point sampling in our own bulk data, there are many interaction QTLs that were not detected in the single-cell data alone, *or* bulk data alone, but enabled by the integration of single cell and bulk data together. We agree that these points were not sufficiently emphasized, and have modified the *Deconvolution* section of the manuscript to highlight the benefits of this analysis.

Unfortunately, identification of genetic determinants of lineage preference, though interesting, was difficult in this data. Generally, it requires a genome-wide association test, for which we are underpowered in this study with only 19 individuals. (Specifically, the multiple testing burden for such a genome-wide search is much greater than required for cis-eQTL calling, where we limit the testing for each gene to a 100kb window around the transcription start site.) Furthermore, recent evidence suggests a significant role of non-genetic factors in determining lineage preference, which would be difficult to resolve with a sample size of 19 individuals (see Jiang et al., *bioRxiv*, 2021. Response to Reviewers Ref. 2). This is an important direction for future study.

The cells are very shallowly sequenced (median UMI appears to be ~1k per cell), and Figure S14 suggests large differences in the number of cells at each sampled time point for each individual. As such, I suspect the point estimates of the binned pseudobulk proportions (ultimately used for differential expression over pseudotime) are highly uncertain, and that downstream differential expression analyses are highly sensitive to the binned pseudobulk library sizes. Specifically, the paper could be strengthened by reporting how reliably expression patterns over pseudotime can be distinguished from random (which could be shown as a supplemental figure). I'd recommend the authors do the following: 1) run 100 random permutations of assigning cells to pseudotime bins 2) run regression for each permutation / gene combination 3) construct empirical distribution of the permuted regression likelihoods for each gene 4) using the gene likelihood under the true pseudotime ordering and the empirical distribution from step 3, report the empirical CDF value 5) report the empirical CDF values for genes as a histogram

We thank the reviewer for this recommendation. We have added the following analysis to the supplement and referenced it in the *Mapping of Dynamic eQTLs* section:

> We also permuted pseudotime across all cells to investigate sensitivity to uncertainty surrounding pseudotime binning and variation in pseudobulk library sizes. For each of 100 cell-level permutations, we aggregated pseudobulk by the permuted pseudotime quantile bins, and performed dynamic eQTL calling. We used the absolute value of the resulting t-statistics to generate an empirical null distribution for each gene. The

empirical p-values generally agreed with the original results up to the significance threshold imposed by the number of permutations performed (p=0.01, -log10(p)=2). These results do not suggest inflation of the nominal p-values from the original analysis, and were in fact less conservative than the p-values obtained using Student's t-distribution (S11 Fig).

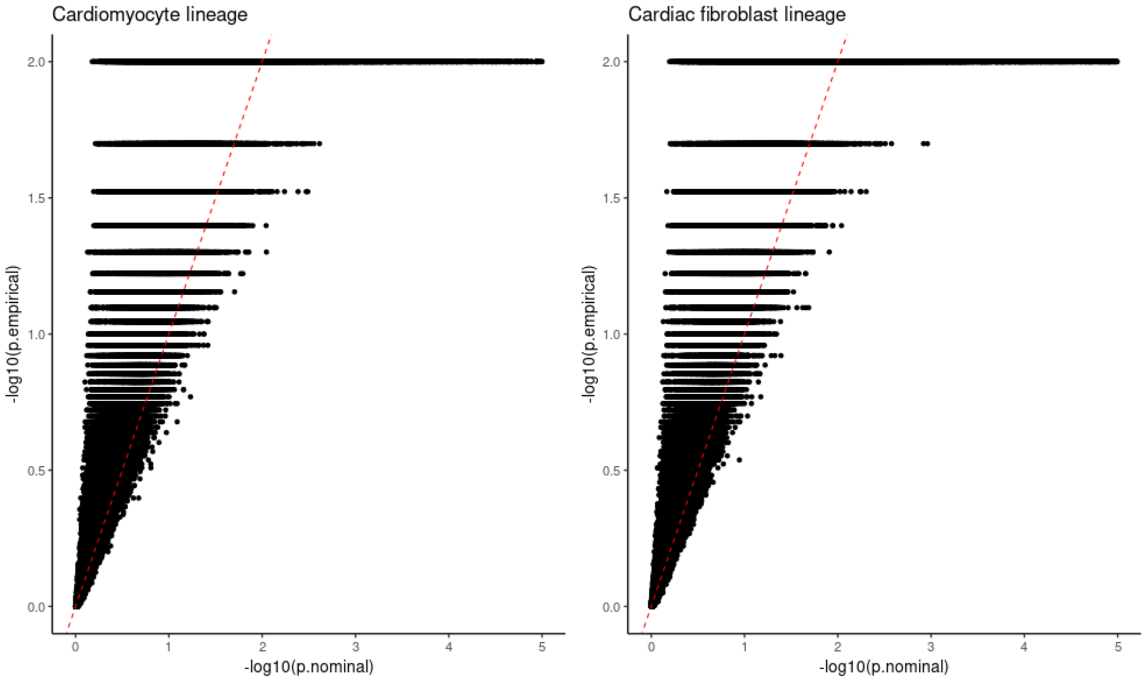

S11 Fig: Cell permutation analyses. We generated an empirical null distribution by performing dynamic eQTL calling after permuting cell pseudotime upstream of pseudobulk aggregation. We compared the resulting empirical p-values (y-axis) to the nominal p-values from the original analysis (x-axis) in both lineages. We did not find evidence of inflation in the nominal p-values from the original analysis (instead, the contrast between the distributions suggests the nominal p-values may be overly conservative).

Don’t find the section title “Single-cell data offers a highly resolved view of cellular differentiation” to be very informative. Would suggest the authors modify the title of this section to more meaningfully reflect its content.

We agree, we have changed this section title to “Single-cell expression data resolves bifurcating trajectories during cellular differentiation”

References

1. Cuomo ASE, Alvari G, Azodi CB, single-cell eQTLGen consortium, McCarthy DJ, Bonder MJ. Optimizing expression quantitative trait locus mapping workflows for single-cell studies. Genome Biol. 2021 Jun 24;22(1):188.

2. Jiang CL, Goyal Y, Jain N, Wang Q, Truitt RE, Cote AJ, et al. Cell type determination for cardiac differentiation occurs soon after seeding of human induced pluripotent stem cells [Internet]. bioRxiv. 2021 [cited 2021 Aug 9]. p. 2021.08.08.455532. Available from: https://www.biorxiv.org/content/10.1101/2021.08.08.455532v1.full.pdf+html
