# Supplementary material for: Single-cell sequencing reveals lineage-specific dynamic genetic regulation of gene expression during human cardiomyocyte differentiation
Source: PLoS Genet. 2022 Jan 21;18(1):e1009666. doi: 10.1371/journal.pgen.1009666 (PMC8809621; doi:10.1371/journal.pgen.1009666)
Supplement: Attachment — Submitted filename: review.docx [file pgen.1009666.s027.docx]

Comments:

- Overall, the manuscript is well written, and information is presented in a logical manner
- Around line 272, the authors comment on the overlap between linear/nonlinear eQTLs identified in the cardiomyocyte lineage with eQTLs identified across all tissues in GTEx. For both the linear and nonlinear eQTLs, this overlap is ~30%. The authors mention (line 260) that nonlinear QTLs can transiently alter expression during differentiation, and thus genetically determined expression differences may not be evident in the terminal cell types. As such, I would anticipate that nonlinear QTLs would be less likely to replicate than the linear QTLs with GTEx. The manuscript could be strengthened by adding a couple sentences in this section discussing the implications of the similar replication rate between the linear / nonlinear eQTLs and why this was observed.
- In the “Mapping of dynamic eQTLs” section, the manuscript could be strengthened by commenting on why there are many fewer dynamic eQTLs for the cardiomyocyte lineage compared to the fibroblasts.
  - Is there a large difference in the number of cells captured in this lineage, and so perhaps this is due to a discrepancy in power?
  - If not due to discrepancy in power, perhaps it reflects that disruptions in expression over fibroblast trajectory are more deleterious to organismal fitness?
- As constructed, the cell type interacting QTLs analyses likely identify QTLs that largely overlap with the pseudotime-based QTLs identified in the previous section, as the proportion of the terminal cell type proportions are highly correlated with their respective pseudotimes. Given this, and given that the cell type interacting QTLs section mainly focuses on the results of the two terminal cell types, this section does not add much to the manuscript. To improve the contribution of this section to the overall manuscript story, one suggestion I have would be to analyze and discuss the genetic determinants of the ratio of the proportions of the terminal cell types (i.e. genetically determined lineage preference).
- The cells are very shallowly sequenced (median UMI appears to be ~1k per cell), and Figure S14 suggests large differences in the number of cells at each sampled time point for each individual. As such, I suspect the point estimates of the binned pseudobulk proportions (ultimately used for differential expression over pseudotime) are highly uncertain, and that downstream differential expression analyses are highly sensitive to the binned pseudobulk library sizes. Specifically, the paper could be strengthened by reporting how reliably expression patterns over pseudotime can be distinguished from random (which could be shown as a supplemental figure). I’d recommend the authors do the following: 1) run 100 random permutations of assigning cells to pseudotime bins 2) run regression for each permutation / gene combination 3) construct empirical distribution of the permuted regression likelihoods for each gene 4) using the gene likelihood under the true pseudotime ordering and the empirical distribution from step 3, report the empirical CDF value 5) report the empirical CDF values for genes as a histogram
- Don’t find the section title “Single-cell data offers a highly resolved view of cellular differentiation” to be very informative. Would suggest the authors modify the title of this section to more meaningfully reflect its content.
